# The socio-economic burden of cystic echinococcosis in Morocco: A combination of estimation method

**DOI:** 10.1371/journal.pntd.0008410

**Published:** 2020-07-31

**Authors:** Aouatif Saadi, Fatimaezzahra Amarir, Hind Filali, Séverine Thys, Abdelkbir Rhalem, Nathalie Kirschvink, Marianne Raes, Tanguy Marcotty, Mohamed Oukessou, Luc Duchateau, Hamid Sahibi, Nicolas Antoine-Moussiaux

**Affiliations:** 1 Fundamental and Applied Research for Animals and Health (FARAH), Faculty of Veterinary Medicine, University of Liege, Belgium; 2 Department of Pathology and Veterinary Public Health, Parasitological Unit, Agronomic and Veterinary Institute Hassan II, Rabat, Morocco; 3 National School of Public Health, Ministry of Health, Rabat, Morocco; 4 Department of Public Health, Institute of Tropical Medicine, Antwerp Belgium; 5 Integrated Veterinary Research Unit, Department of Veterinary Medicine, University of Namur, Belgium; 6 Department of Veterinary, Biological and Pharmaceutical Sciences, Agronomic and Veterinary Institute Hassan II, Rabat, Morocco; 7 Faculty of Veterinary Medicine, Department of Nutrition, Genetics and Ethology, Gent, Belgium; University of Zurich, SWITZERLAND

## Abstract

Cystic echinococcosis (CE) is a major zoonosis in Morocco despite the launch of a national control programme in 2005. As its economic consequences have not been studied yet in Morocco, this study estimated CE impact in terms of monetary losses, disability-adjusted life years (DALY), and DALY for zoonotic diseases (zDALY) in the entire country and in specific regions for the 2011 to 2014 period. The direct monetary losses were related to organ seizure from infected animal in slaughterhouses, and to healthcare expenses as well as lost wages for infected humans. Animal production losses concerned milk yield, fertility, carcass weight, and wool production. Losses due to human infection were also composed of disability and productivity losses at work. Monte Carlo simulations were used to estimate monetary losses and zDALY values. Nationwide, the estimated DALY was 0.5 years per 100,000 persons per year, and the zDALY was 55 years per 100,000 persons per year. Total yearly losses were estimated at 73 million USD (54–92 million USD). However, losses differed significantly among regions. Most of the economic losses consisted of unperceived consequences, i.e. decreased animal production and reduced productivity of asymptomatic individuals. Future studies should determine the socioeconomic and epidemiological factors underlying the differences in economic losses among regions to develop better adapted control programmes.

## Author summary

Cystic echinococcosis (CE) is a major neglected zoonosis in Morocco, despite the launch of a national control programme in 2005. The first study on CE in Morocco dates back to 1924. However, no evaluation of economic losses was made until now. The present study estimated the economic losses caused by CE in Morocco, at the national and regional scale, by combining financial and non-financial methods. Estimation of the direct and indirect losses caused by CE infection in humans and livestock (sheep, cattle, goats and camels) highlighted the important disease burden nationwide, amounting to 0.07% of Morocco Gross Domestic Product. The combination of methods brought information on the different CE-linked economic losses, including the unperceived consequences. These results indicate that the national CE control strategy did not result in a decrease of the disease burden, which calls for its evaluation and improvement.

## Introduction

Cystic echinococcosis (CE) is a parasitic zoonosis caused by *Echinococcus granulosus*. The cycle includes canids as definitive host, and herbivores and omnivores as intermediate hosts. Intermediate hosts are infected through the ingestion of *E*. *granulosus* eggs released in canids’ faeces. Then, cysts are formed in various organs of the intermediate host, particularly liver and lungs. Canids are infected by consuming infected intermediate hosts with cysts, and will develop the adult form in their intestinal tract. Humans are an accidental intermediate host. The risk is particularly high for rural populations, where dogs are kept for herding and people have low standards of living. CE represents a public health issue worldwide [1].

In Morocco, CE in humans is a notifiable disease. However, the surveillance system, supervised by the Ministry of Health [2], is mainly based on recording the patients undergoing surgery for hydatid cysts in public hospitals [3]. For the periods 1980–1992 and 2003–2008, 23,512 patients who underwent surgery for hydatid cysts were recorded in the Ministry of Health registry, and the mean annual incidence of surgical cases increased from 3.6 to 5.2 per 100,000 persons from 1980 to 2008 [4]. The most affected regions are Meknes-Tafilalt and Chaouia-Ouardigha [4]. More recent data provided by the ministry of Health on the annual incidence of human CE reported 7,536 operated cases for the period from 2009 to 2014, and the mean annual incidence of surgical cases decreased from 5.3 to 2.2 per 100,000 person from 2009 to 2014. However, ministerial data are underestimating the true prevalence of infection, because many infected individuals do not present symptoms, or because part of people with symptomatic disease will not be treated by surgery, and therefore will not be recorded in the Ministry of Health registry [5]. Almost all hydatid cysts reported in Morocco (95%) are diagnosed by ultrasound examination [6] and the studies on CE prevalence in humans are rare and are often focused on rural areas [7]. In 2014, an ultrasound screening in the Mid Atlas reported that the prevalence of abdominal CE was 1.9%. Moreover, 52.1% of them were asymptomatic [5]. The World Health Organization Informal Working Group on Echinococcosis (WHO-IWGE) implemented a classification in clinical categories to guide the management of patients with CE (surgery, medical therapy, percutaneous treatment, and “watch and wait” approach) [8]. However, in Morocco, CE treatment is almost always surgical, while other options, such as percutaneous treatment, are rare [4].

Data on animal CE in Morocco come from records established by the veterinary services of slaughterhouses, CE being also a notifiable disease in animals [3]. For the period 2001–2004, CE prevalence based on this post-mortem inspection was estimated at 23.0% in cattle, 12.0% in camels, 10.6% in sheep, and 1.9% in goats [9]. Data for 2014 provided by the Ministry of Agriculture show that CE prevalence at slaughterhouses was 12.4% in cattle, 8.7% in camels, 8.4% in sheep and 4.7% in goats. In all species, infection is reported more frequently in older animals [9,10]. This could be mainly explained by the cumulative exposure risk, as well as the time required for the development of hydatid cysts. Indeed, smaller microscopic and early cysts may escape inspection [10]. Therefore, data from slaughterhouses would underestimate the true CE incidence in farm animals throughout Morocco, because young animals are more frequently slaughtered [11]. Moreover, animals slaughtered at home, a common practice in Morocco, and animals slaughtered during the feast of sacrifice (i.e., 5 million small ruminants, one per household) are consumed without any sanitary control [12].

In 2004, Morocco has set up a national programme of CE control by creating an inter-ministerial CE control committee that involved the Ministry of Agriculture, the Ministry of Health and the Ministry of the Interior. In 2005, a monitoring guide was developed and the programme was launched in 2007, when CE notification became mandatory [3]. This programme is based on three main strategic axes: i) in animals, stopping the parasite life cycle, protecting the livestock, and controlling the dog population; ii) in humans, early detection and treatment of individuals with hydatid cysts; and iii) elaboration of an appropriate legislative and regulatory arsenal [3]. The aim of this program was to decrease by 50% the incidence in human (2.8 cases per 100,000 person in year) [3]. However, the implementation of integrated control measures has been extremely difficult due to inter-ministerial collaboration issues, and only the notification system of patients undergoing surgery for CE has been put in place, which does not fit the goal of early detection [5]. Moreover, no official evaluation of the program has been carried out yet. The causes of CE persistence in Morocco are manifold, including the slaughterhouse practices, and the poor understanding of the parasite life cycle by the population that leads to risky behaviors [13,14]. Indeed, the slaughterhouse infrastructure and their practices are unsatisfactory, and dogs (owned and stray) have access to infected organs [13,15]. A study conducted in the Middle Atlas showed a high prevalence of CE infestation in dogs, ranging from 23.5% to 38.8% in owned dogs and from 51.3% to 68.5% in stray dogs [16].

Due to the high disease burden still observed despite this national programme, corrective policies and actions are needed. In this context, precisely assessing CE socio-economic impacts is essential [17]. Indeed, CE burden is important due to its morbidity, mortality, and socio-economic losses [18]. This can be estimated using two main methods: disability-adjusted life years (DALY) and monetary losses [19]. Monetary losses concern humans (healthcare expenses, loss of vitality and health [20]) and livestock (organ seizure and reduced animal production in terms of carcass weight, milk yield, fertility, and wool [21]). For example, a study conducted in Turkey showed that the presence of hydatid cysts reduced the average carcass price by 4.4% [22]. CE economic importance in slaughtered animals results from the seizure of liver, lungs or any other infected organ, sometimes even the whole carcass [23].

DALY is a non-financial method to estimate disease burden in humans, and is considered the reference disease-burden metric by the WHO [18]. DALY calculation aggregates the loss in healthy life years by considering the adjusted life years lived with disability (YLD) and the years of life lost due to premature mortality (YLL) [24]. However, in the case of zoonosis, DALY does not take into account the burden caused by animal infection [25]. Therefore, the method has been modified for zoonotic diseases, estimating the so-called zDALY [17]. Specifically, this metric includes also the animal loss equivalents (ALE) that is calculated by quantifying the livestock losses divided by the national income value. ALE reflects the “labour time lost” due to a zoonotic disease [26].

In the prospect of informing future policies, the aim of the present study was to use financial and non-financial methods to estimate CE burden in Morocco, at national and subnational scales, to enable public health policy-makers to optimally allocate the limited resources and to design effective control campaigns.

## Material and methods

### Ethics statement

All data used in this study were in the form of aggregated data that cannot be associated with any specific individual. Therefore, neither consent nor ethical clearance was needed for this study.

### Study area

**Fig 1:**
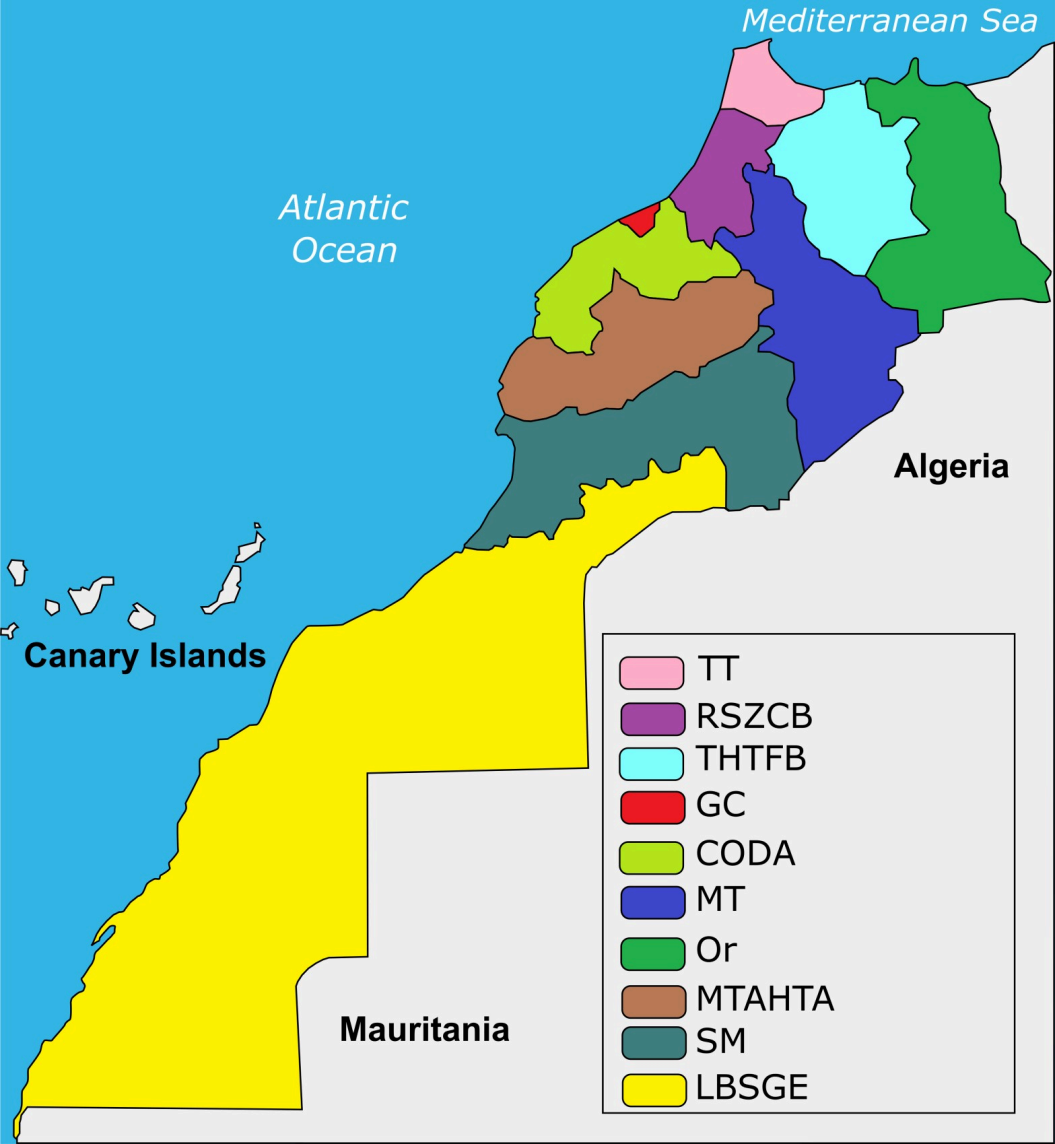
Regions of Morocco according to ONSSA. CODA: Chaouia Ouardigha Doukkala Abda; GC: Grand Casablanca; LBSGE: Laayoune Boujdour Sakia El Hamra Guelmim Essmara; MT: Meknes Tafilalet; MTATA: Marrakech Tensift Al Haouz Tadla Azilal; Or: Oriental; RSZCBH: Rabat Sale Zemmour Zaer Chrarda Bni Hssen; SM: Souss Massa Draâ; THTFB: Taza Alhoceima Taounate Fes Boulemane; TT: Tanger Tetouan (Adapted from Wikimedia commons: https://commons.wikimedia.org/wiki/File:Morocco_Regions_97-11_numbered.svg).

This study covered the entire territory of Morocco, and included also a comparison of its regions, as defined by the National Office for Food Safety (ONSSA) (Fig 1), because all data provided by the different ministries are presented by region. Moreover, data were also collected in Khénifra (Meknes Tafilalet region), the city with the highest CE incidence in humans in Morocco.

For this study, which was started in 2016, data for the 2011 to 2014 period were collected because they were available in digital format for all regions.

#### Economic losses for people with CE

**Fig 2:**
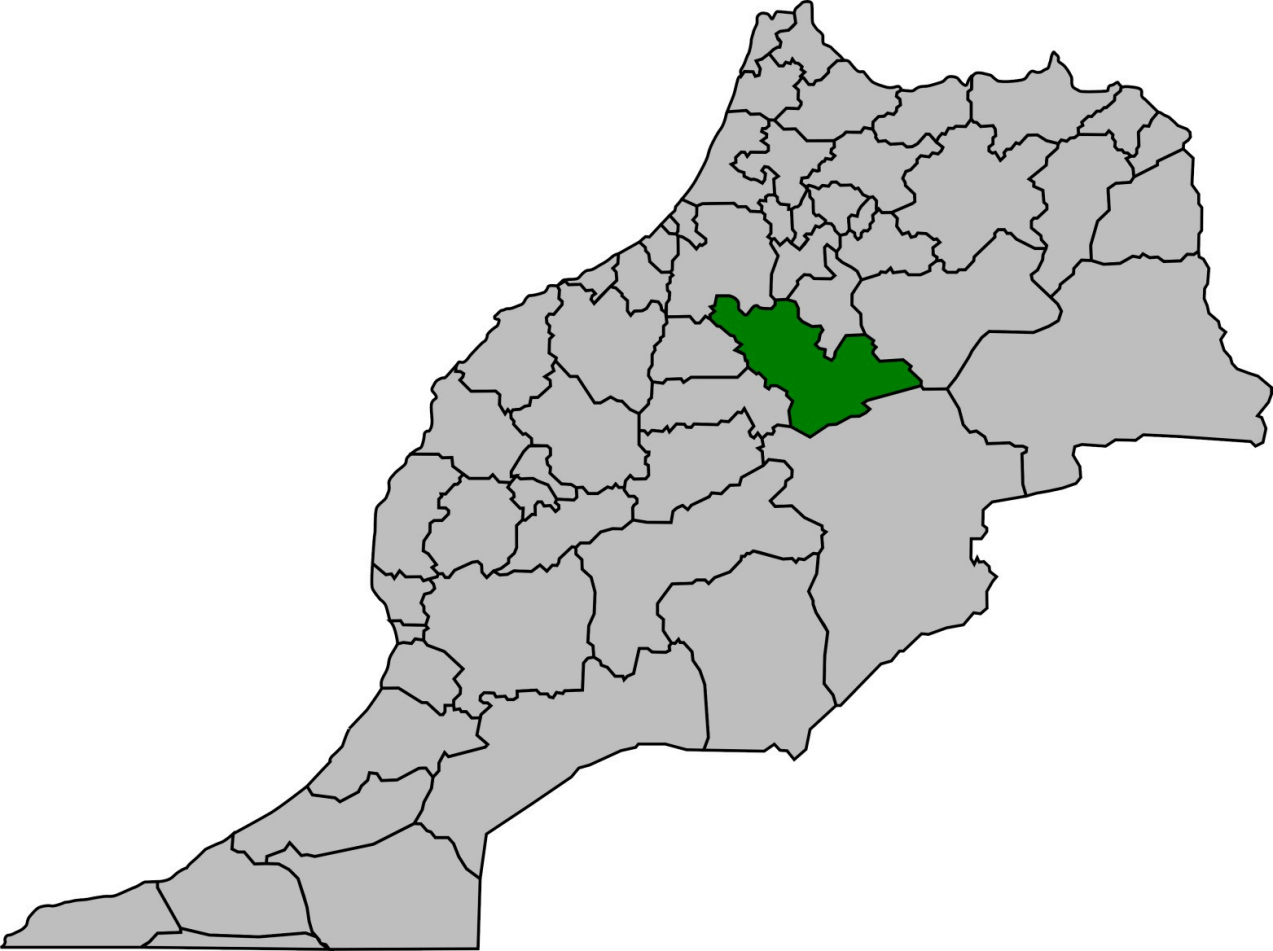
Khénifra province (in green). (Source: Wikimedia commons: https://commons.wikimedia.org/w/index.php?search=province+de+khenifra&title=Special%3ASearch&go=Continuer&uselang=fr&ns0=1&ns6=1&ns12=1&ns14=1&ns100=1&ns106=1#/media/File:Khenifra_in_Morocco.png).

This study estimated the economic losses for people who underwent surgery for CE and also for asymptomatic people. The Ministry of Health provided the incidence of patients with CE who underwent surgery in public-sector hospitals per region and per year. Only the costs related to surgical treatment was estimated because CE treatment in Morocco is almost only surgical [4]. Losses for asymptomatic undiagnosed people were based on productivity losses. Data on different surgical interventions cost were extracted from the records of the hospital of Khénifra (capital of Mid Atlas) (Fig 2). To calculate the additional costs related to hospitalization (travel costs, food, etc.), patients who underwent surgery at Khénifra hospital (n = 14) were contacted and asked to fill in a questionnaire (Table 1).

**Table 1:** Questionnaire sent to patients with CE who underwent surgery at Khénifra hospital to determine the additional costs.

Number of accompanying persons Transport cost per person Cost of lost work days for the accompanying person(s) Costs before surgery -Laboratory/imaging analyses -Drugs -Diagnosis-related exams - Lost income

In Morocco, 84% of hydatid cysts are localized in liver and 12% in lungs [4], followed by kidney, peritoneum, spleen and pancreas, which are included in the abdominal surgery category [6]. Therefore, our estimates were based on the fact that 88% of patients had abdominal surgery and 12% had thoracic surgery. The cost related to hospitalization covered medical fees (e.g. drugs, diagnosis-related exams, and surgery), non-medical expenses (e.g. accompanying family member, transportation, accommodation and food), workdays lost before hospitalization, and additional costs for postoperative complications, if applicable. Because of the absence of post-surgery course information, the post-surgery costs were based on literature data indicating the absence of post-surgery complications for 97.06%, post-surgery complications for 0.12%, recurrent illness for 0.31%, and death for 0.2% of patients who undergo surgery for CE in Morocco [6].

According to Khénifra hospital records, the mean hospital stay was 8.15 days (1–34 days), and the post-surgery leave prescribed ranged from 10 to 30 days. The estimated loss of work days ranged from 1 day to 1 year in the event of death [27]. A loss of 100% of daily wages was assumed for patients during the post-surgery leave. In the case of unemployed people, the loss of workdays was equal to zero. The unemployment rate was 15.2% for men and 30.5% for women in urban areas, and 8.3% for men and 26.2% for women in rural areas [28]. CE frequency was 62.52% in rural areas and 37.48% in urban areas [6]. The loss of work days was estimated from the gross national income (GNI) per capita in Morocco that shows drastic differences between women and men [29]. Given the absence of data on GNI per capita by gender, this difference was estimated from data published by the United Nations Development Program (UNDP). In 2013, the estimated GNI per capita in Morocco, based on purchasing power parity (PPP) (Constant 2011 PPP USD), was 10,692 USD for men and 3,215 USD for women [30,31]. Therefore, the GNI per capita was 46.56% for women and 154.84% for men of the GNI per capita in Morocco (i.e. 6,905 USD) [32].

**Fig 3:**
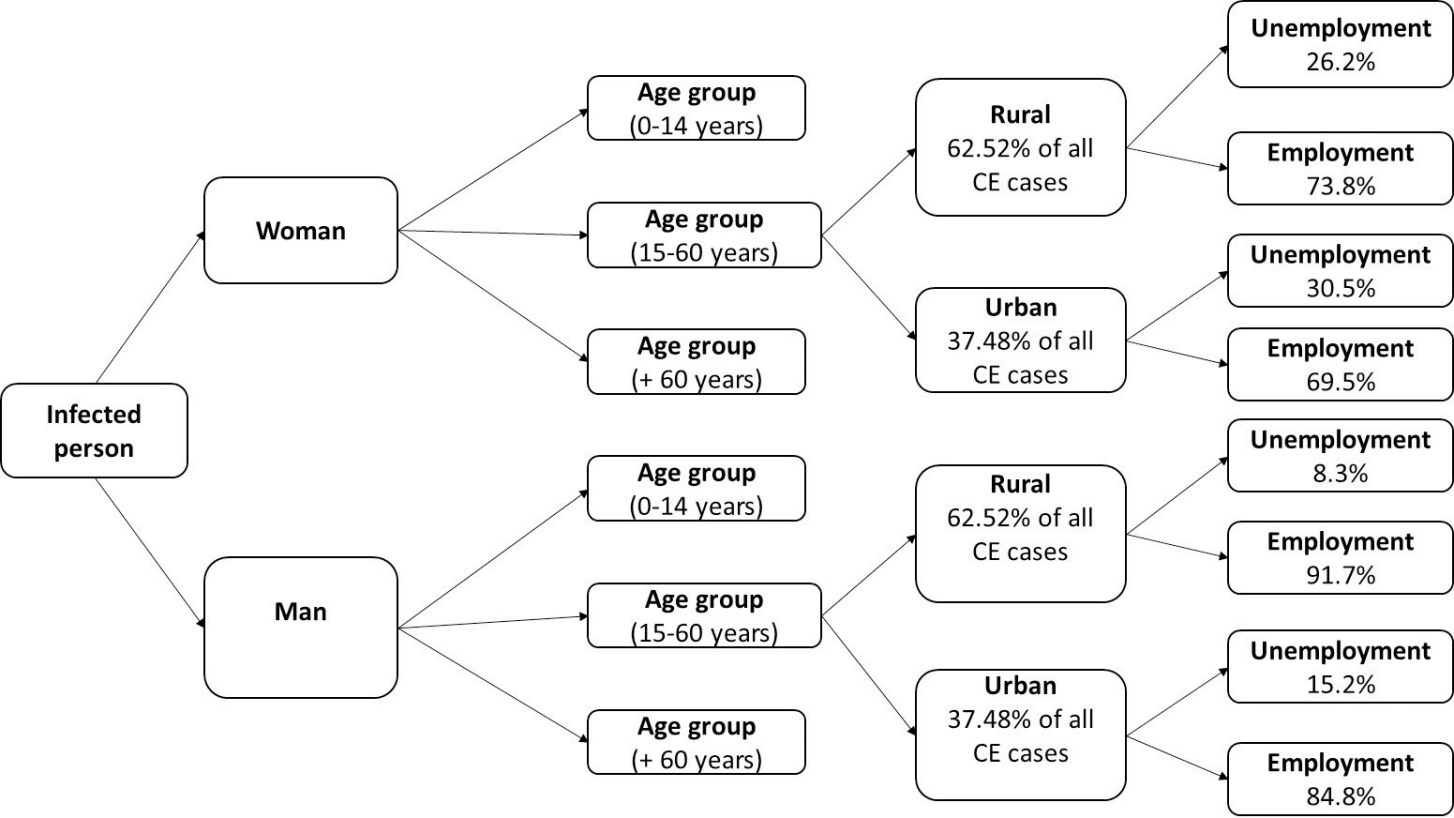
Schematic description of how the loss of workdays was estimated in function of the patient age group and environment features.

When the operated patient was a child or an elderly person, a loss of 30% of the income of one relative was applied for the hospitalization period, based on the assumption that one family member devoted part of his/her time to take care of the hospitalized person [21,27]. In Morocco work starts from the age of 15, therefore, the 15–60 age group was considered the working age group [33]. Fig 3 presents the diagram used to estimate the GNI loss per capita. The distribution of cases by age and gender was estimated using data extracted from the Khénifra hospital records.

The loss of productivity by patients undergoing surgery for CE and asymptomatic individuals was estimated from literature data at 2%, with a uniform distribution from 0% to 4% [34,35]. Being undiagnosed, this number of cases is not known in Morocco. Therefore, the number of undiagnosed cases was estimated using the available data on abdominal CE from the ultra-sound screening-based study by Chebli and co-workers in 2017, who found a prevalence of 2.6% in Ifrane and 1.3% in El Hajeb (Mid Atlas) [5]. No corresponding data could be found for pulmonary cases in Morocco nor in neighboring countries. The incidence of patients with CE who underwent surgery, recorded by the Ministry of Health for the same period, was 18 in Ifrane and 10 in El Hajeb. The prevalence of asymptomatic cases was then estimated as follows:

$$Prevalence\;of\;asymptomatic\;cases=\left[\begin{array}{c} \frac{(Prevalence*Observed\;population)-Surgical\;Incidence}{Observed}\\ \mathrm{population} \end{array}\right]*100$$

and ranged from 1.9% to 2.2%. Due to the uncertainty associated with this estimate, a triangular distribution was used, with a minimum value of 0%, a maximum value of 2.2%, and a probable value of 2.05% (the mean of the extrapolated values). The same diagram presented in Fig 3 was used to estimate the productivity losses linked to asymptomatic CE, by taking into account age (15 to 60 years), gender (man/woman), area (rural/urban), and unemployment rate.

#### Livestock losses due to CE

The species that were considered here are sheep, cattle, goats and camels (i.e. the most common species in the country). CE prevalence, organ or carcass seizure due to CE, number of slaughtered animals, and number of animals by sex and age were provided by ONSSA and HCP. Data on the national production of milk, meat and wool were supplemented with data from the World Bank website. The CE-linked yield reduction rates (Table 2) used for the calculation of each parameter were obtained from literature data [36–39]. The price of milk was provided by the largest milk collector in Morocco (Centrale laitière). The Khénifra Butchers Union provided the mean price of meat and offal (liver and lung). The two largest Moroccan associations of sheep and cattle farmers provided the prices of new-born calves/lambs and wool.

**Table 2:** Percentage of animal product reduction caused by CE.

Parameter	Reduction rate (%)	Source
**Cattle**		
Meat	2.5–10%	[36]
Milk	2.5–5%	[36]
Fertility	9.9–12.1%	[36]
**Sheep**		
Meat	5–20%	[36,37]
Wool	10–40%	[36,37]
Fertility	9.9–12.1%	[36,37]
**Goats**		
Meat	5–20%	[37]
Fertility	9.9–12.1%	[37]
**Camels**		
Meat	2.5–10%	[38,39]

Table 3 summarizes the formulas used to estimate animal production losses. Specifically, offal losses were calculated for two organs (lung and liver: the most frequently seized organs). For cattle, sheep and goats, the percentage of lung and liver seized was extrapolated from the Khénifra slaughterhouses’ records. For camelids, data were retrieved from a study on CE prevalence in camels in Mauritania [40], a country that borders the Moroccan region where camel farming is concentrated. The average production of each parameter per animal (milk, wool, meat and new-born) was estimated by dividing the type of animal production on the number of animal concerned.

**Table 3:** Formulas used to estimate animal product losses (milk, wool, meat and fertility).

Type of product	Formula
Percentage of offal losses	• Total offal losses^(##)^ = liver losses + lung losses • Percentage of liver losses^(##)^ = (liver losses/total offal losses)*100 • Percentage of lung losses^(##)^ = (lung losses/total offal losses)*100
Production	• Average weight per carcass^(^)^ = annual carcass production/number of animals slaughtered in that year • Average annual milk yield per cow^(^)^ = total milk production of the country/number of dairy cows • Average annual wool production^(^)^ = total wool production in the country/number of sheep • Mean number of new-borns per female^(^)^ = total number of births/number of breeding females
Monetary losses due to offal losses	• Liver losses = (total seizures due to CE^(#)^ * Percentage of liver losses)* price per kg of liver • Lung losses = (total seizures due to CE^(#)^ * Percentage of lung losses)* price per kg of lung • Total losses = Liver losses + Lung losses
Meat losses	• Weight losses = ((Average carcass weight × percentage of carcass weight reduction^(###)^)* number of infected animals^(#)^)* price per kg of meat
Milk losses	– Number of infected cows = number of dairy cows^(#)^ * prevalence of CE^(#)^ • Milk losses per cow = production of milk per cow * rate of reduction in milk production due to CE^(###)^ • Total milk losses = (number of infected cows * losses in milk per cow)* milk price per litre
Wool losses	• Infected sheep = number of sheep^(#)^ * prevalence of CE • Wool losses per animal = average wool production per animal * percentage of wool production reduction^(###)^ • Total wool losses = (infected sheep * losses of wool per animal)* price of wool
Decreased fertility	• New-born losses = (((number of breeding females^(#)^ * CE prevalence) * average number of new-borns per female) * percentage of fertility reduction^(###)^) * New-born price
(#): ministerial data; (##): estimated from Khénifra records; (###): literature data; (^): estimated from ministerial data	

For the young animals, the prevalence of CE infection corresponds to the prevalence of slaughterhouses. For the adult animals (sheep and goats over 2 years old, and cows over 3 years old), analyses were carried out using a minimum prevalence that corresponded to CE prevalence at slaughterhouses, and a maximum prevalence from literature data (56% in cattle, 40% in sheep, 20% in camels and 7% in goats) [9]. To estimate the average weight per carcass, which relates to the quantity of meat lost and therefore to slaughtered animals, the prevalence of CE at slaughterhouses was used. Milk losses were only established for cattle and wool losses were estimated for sheep, because other species are not significantly exploited for milk and wool production in Morocco. Losses associated with decreased fertility were estimated as the number of lost new-borns, which are sold at birth. Decreased fertility was not estimated for camels due to the lack of data.

### DALY and zDALY

The DALY represents the sum of the YLL and of the YLD values. In the zDALY, ALE is added to the DALY value [17]. ALE is the time lost to compensate for the monetary losses of livestock (i.e. the number of years of individual work needed to produce the amount lost).

#### DALY estimation

In accordance with other studies, YLL and YLD were considered as resulting only from surgical cases, and asymptomatic cases were considered as not having any significant impact on these two dimensions [19]. DALY was calculated as follows [19,41]:

$$-\left[\frac{DCe^{-\beta a}}{{\left(\beta +r\right)}^{2}}\left[e^{-(\beta +r)(L)}(1+(\beta +r)(L+a))-(1+(\beta +r)a)\right]\right]$$

Where **D** is the disability weight, **C** the age weighting correction constant (C = 0.16243), **β** the age weighting parameter (β = 0.04), **r** the discount rate (r = 0.03), **a** the age at disease onset, **L** the disability duration or time lost due to premature death, and e = 2.71. [19]. In the absence of complications, D = 0.200 and L = 1 year; in the case of postoperative complications, D = 0.239 and L = 5 years; in the case of recurrent illness, D = 0.809 and L = 5 years; and in the case of death after surgery, D = 1 and L = 7.25 [1]. The age at disease onset (a) was extrapolated from the Khénifra hospital records and corresponded to the age at surgery. L was estimated from the literature [1] due to the absence of data.

#### ALE estimation

ALE was estimated by dividing the monetary value of livestock losses by the GNI per capita [17] obtained from the World Bank database.

#### Conversion of total economic losses into DALY-equivalent

To allow for comparisons of the actual DALY with a DALY-like value including the productivity losses for asymptomatic CE patients, a conversion was proposed applying the same calculation as that performed to convert animal economic losses into ALE. Hence, the total annual economic losses caused by CE in humans and animals was converted into lost years by dividing the monetary value of these losses by the GNI for the corresponding year.

### Data analysis

To account for the uncertainty of the proposed estimates, stochastic methods were used for the overall calculations. The 95% confidence intervals for the total loss were computed by running 100,000 Monte Carlo simulations with the Python Programming Language (version 2.7) and by taking the minimum, maximum, mean, median, the 2.5–97.5 percentiles and standard deviation (SD). The choice of distributions was based on the literature, as already detailed here above for several parameters [38]. Regarding CE prevalence for young animals, a beta probability distribution was chosen, with alpha and beta parameters based on the slaughterhouse data in the different regions of Morocco. Alpha was equal to the number of CE cases + 1, and beta to the number of all examined animals minus the number of CE cases + 1. For adult animals, a uniform distribution was used, with a minimum value that corresponded to the prevalence in the slaughterhouses, and a maximum value that corresponded to CE prevalence in old animals in the literature [9]. Triangular distributions were used for product loss parameters (i.e. fertility, carcass weight, meat price, milk production, milk price, offspring price, wool production, wool price).

## Result

### Economic losses

**Fig 4:**
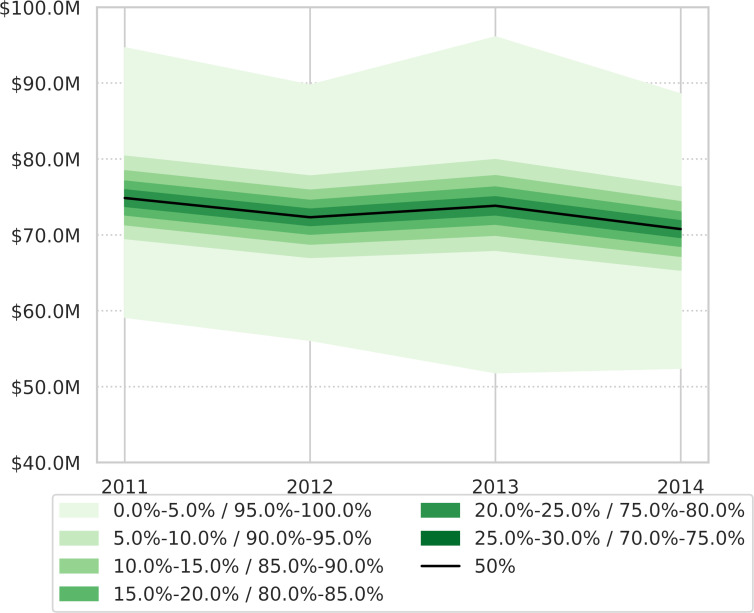
Total economic losses (whole country) caused by CE in humans and animals for the 2011–2014 period. Monte Carlo simulations.

**Fig 5:**
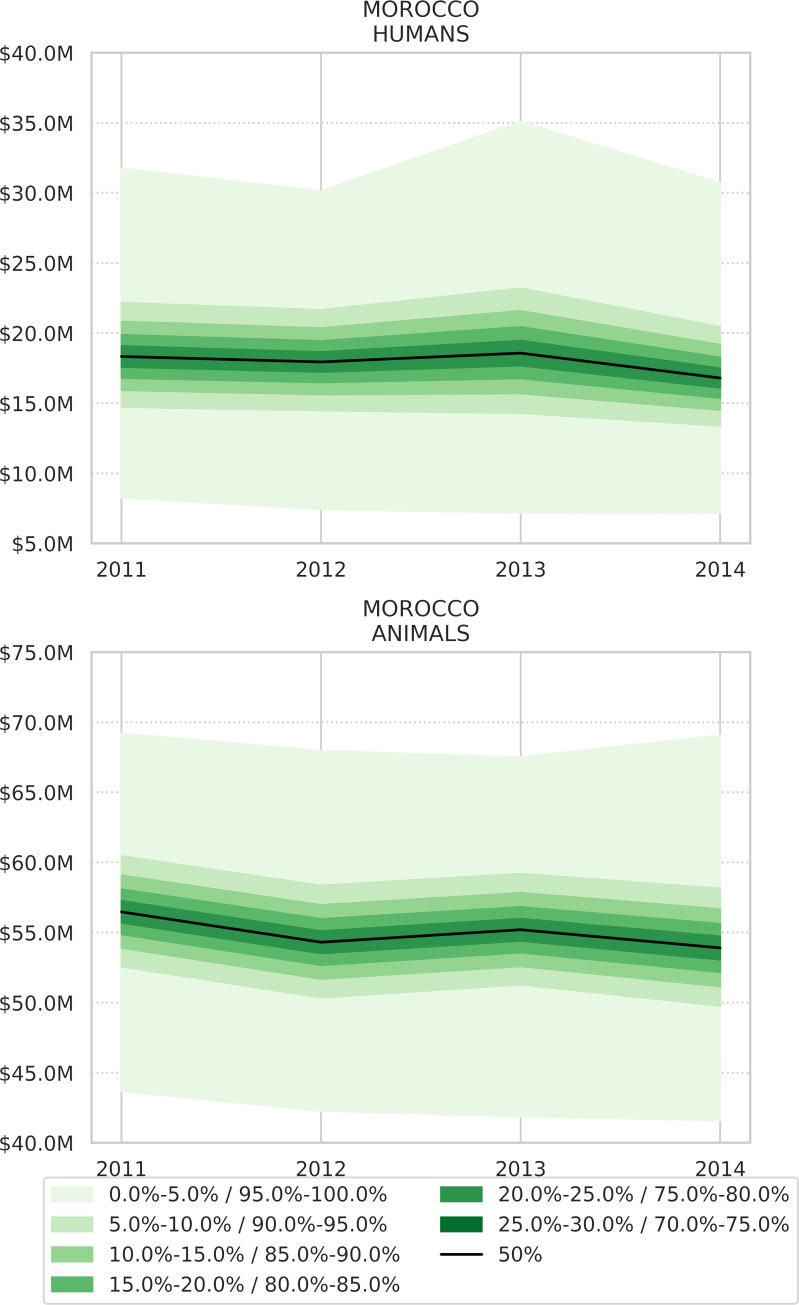
Economic losses (in million USD) for the whole country due to CE in humans (top) and livestock (bottom) for the 2011–2014 period. Monte Carlo simulations.

**Fig 6:**
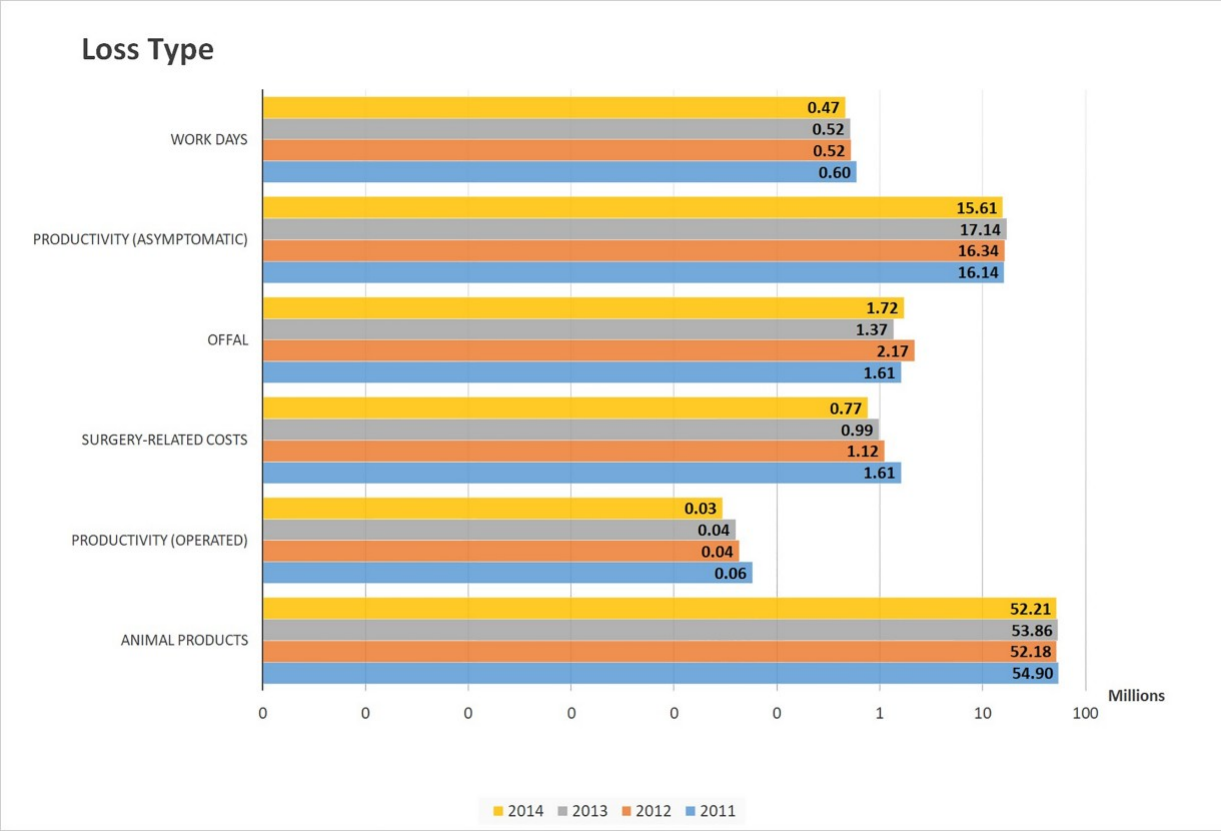
CE-linked economic losses according to the loss type (million USD) for the whole country, logarithmic scale. Monte Carlo simulations. Losses related to surgery include medical and non-medical costs (transport, food, accompanying person…).

All epidemiological parameters are presented in additional files. For the 2011–2014 period, the estimated mean for annual total economic losses caused by CE ranged from 70 to 74 million USD (73 million USD per year on average) (Fig 4; Table 4). The mean of maximal total range (min-max) obtained through Monte Carlo simulations went from 54 to 92 million USD per year. The average loss represented between 0.07% and 0.06% of Morocco annual Gross Domestic Product (GDP) (Table 4). Mean annual economic losses linked to human CE ranged from 16 to 18 million USD per year and that from animal infection ranged from 53 to 56 million USD per year (Fig 5). Most losses were caused by productivity reduction in undiagnosed people and by animal product losses (milk, wool, meat, and fertility) (Fig 6).

**Table 4:** Annual economic losses for the entire country caused by CE and percentage of lost GNI and GDP.

Year	Morocco GNI (million USD)	Total losses (USD)	% lost GNI	Morocco GDP (billion USD)	% lost GDP
2011	99,886.56	74,917,890.71	0.075%	101.370	0.073%
2012	100,417.88	72,367,275.25	0.072%	98.266	0.073%
2013	105,816.42	73,916,607.47	0.069%	106.826	0.069%
2014	106,120.31	70,806,534.55	0.066%	109.881	0.064%

**Fig 7:**
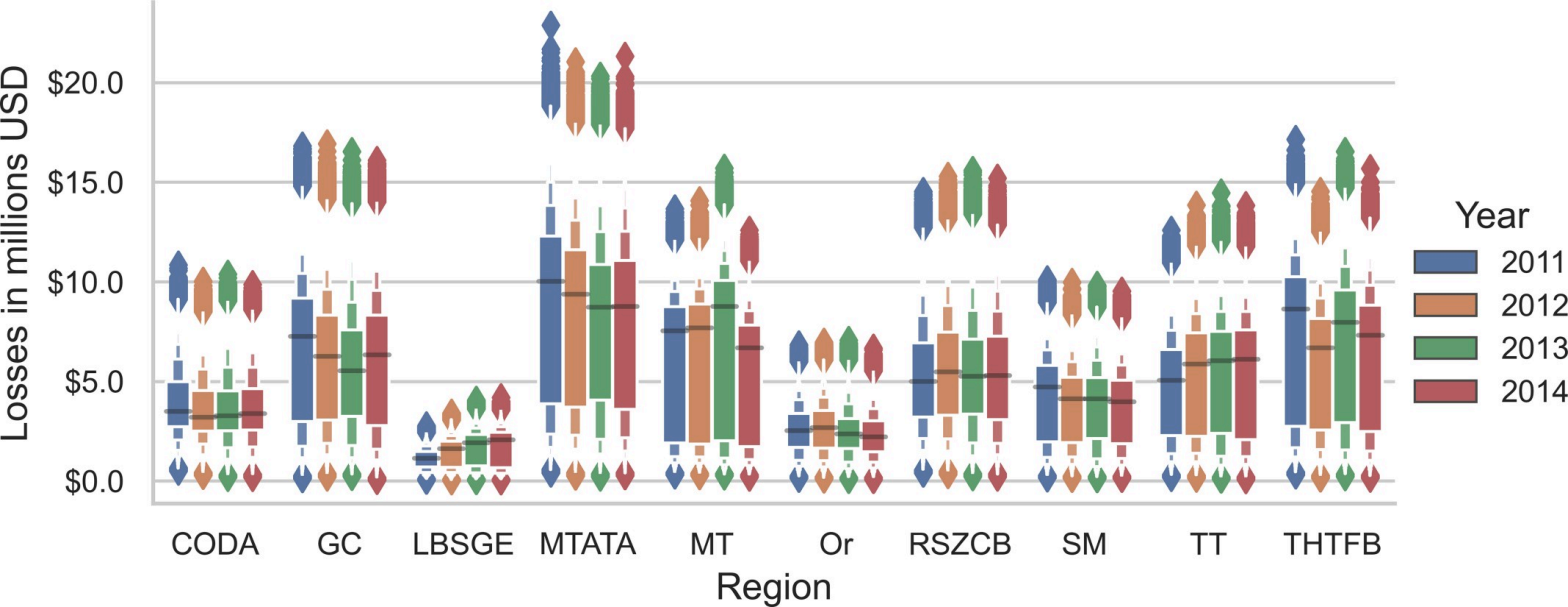
Economic losses (Y-axis = million USD) caused by CE by region estimated with Monte Carlo simulations. .CODA: Chaouia Ouardigha Doukkala Abda; GC: Grand Casablanca; LBSGE: Laayoune Boujdour Sakia El Hamra Guelmim Essmara; MT: Meknes Tafilalet; MTATA: Marrakech Tensift Al Haouz Tadla Azilal; Or: Oriental; RSZCB: Rabat Sale Zemmour Zaer Chrarda Bni Hssen; SM: Souss Massa Draâ; THTFB: Taza Alhoceima Taounate Fes Boulemane; TT: Tanger Tetouan.

**Fig 8:**
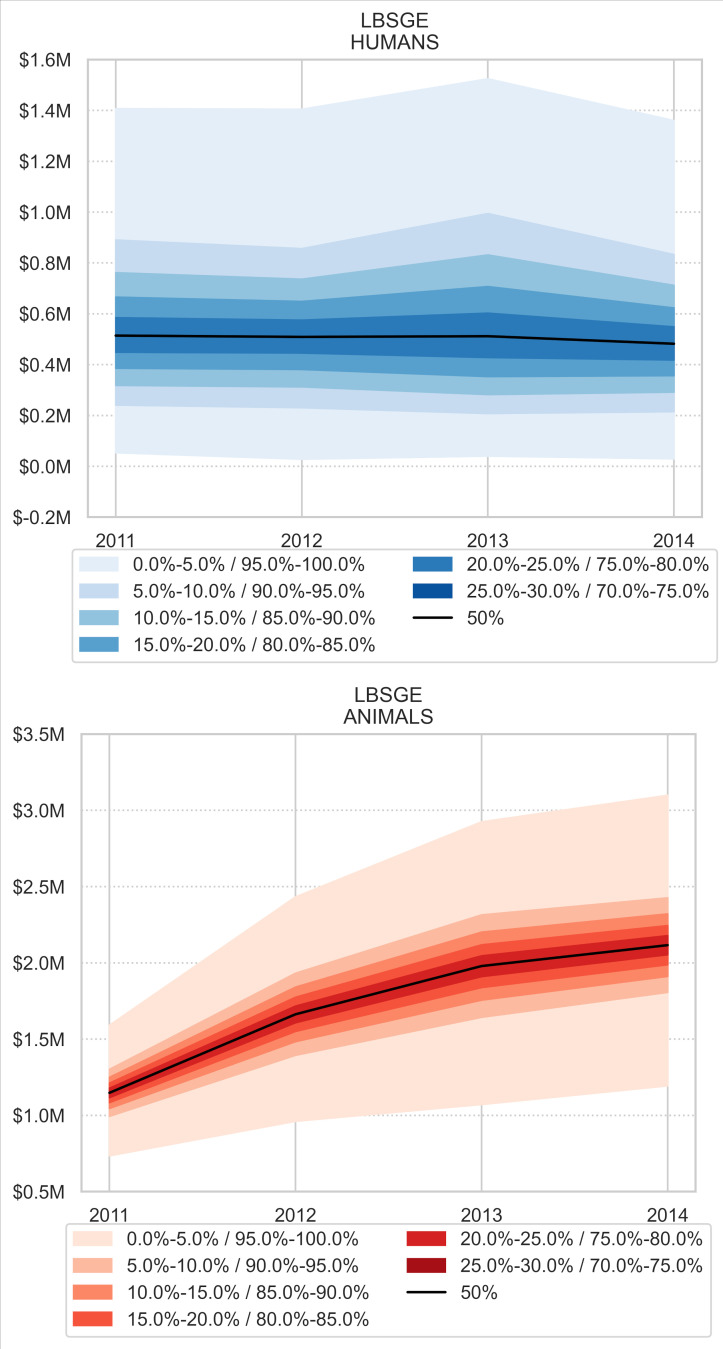
Economic losses (million USD) due to CE in humans (top) and animals (bottom) in the Laayoune Boujdour Sakia El Hamra Guelmim Essmara (LSBGE) region. Monte Carlo simulation.

In most Moroccan regions, losses varied from year to year, but no significant trend of loss reduction was observed in any region from 2011 to 2014 (Fig 7). Marrakech Tensift Al Haouz Tadla Azilal (MTATA) and Meknes Tafilalet (MT) were the regions with the highest economic losses. The province of Khénifra is part of the MT region. Laayoune Boujdour Sakia El Hamra Guelmim Essmara (LSBGE) has the lowest losses, but losses tended to increase from 2011 to 2014, especially losses caused by CE in animals (Fig 8).

### DALY and zDALY

**Fig 9:**
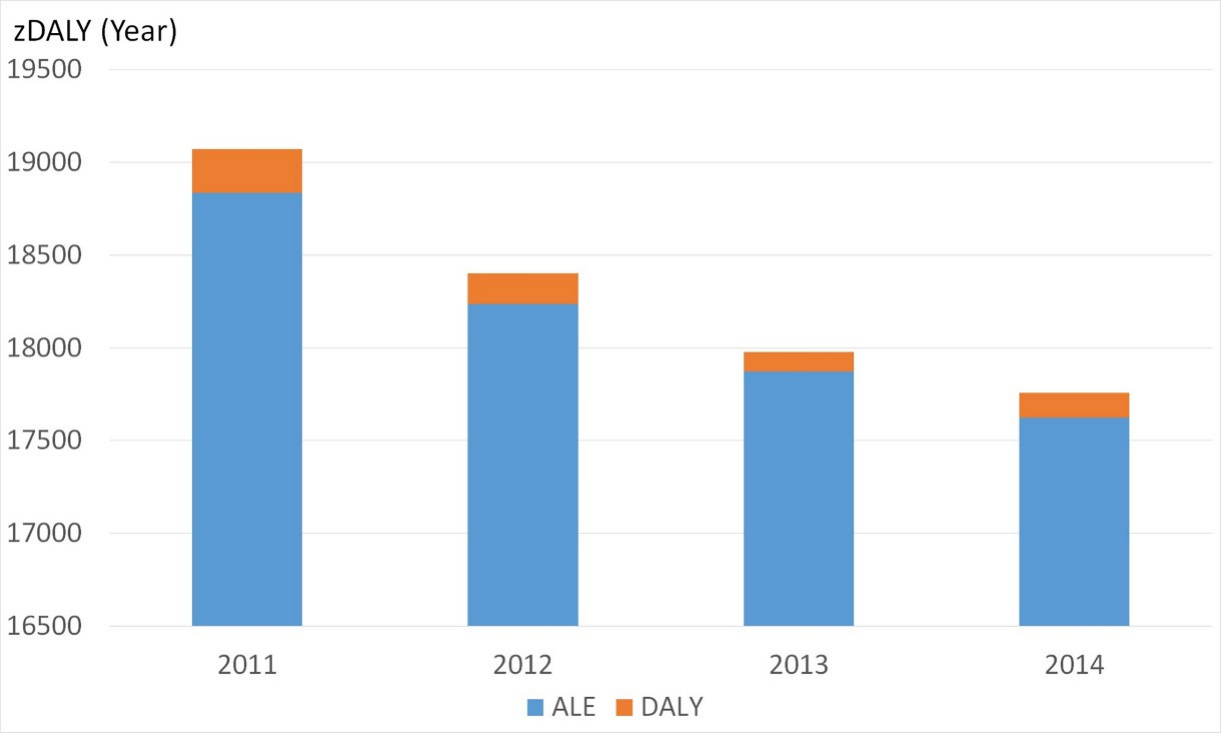
Estimated number of zDALYs per year due to CE in Morocco. The zDALY was calculated by adding the animal loss equivalents (ALE in blue) to the disability-adjusted life years (DALY in orange) value.

The mean (min-max) estimated DALY per year at the national level was 160 years (106–238) (i.e. 0.5 years per 100,000 persons). The mean (min-max) estimated zDALY per year was 18,330 years (17,775–19,074) (i.e. 55 years per 100,000 persons). The DALY, which does not include productivity losses for asymptomatic human cases, represented approximately 0.87% of the total zDALY (Fig 9). ALE, which includes animal productivity losses, thus accounted for almost all of the estimated zDALY value.

**Fig 10:**
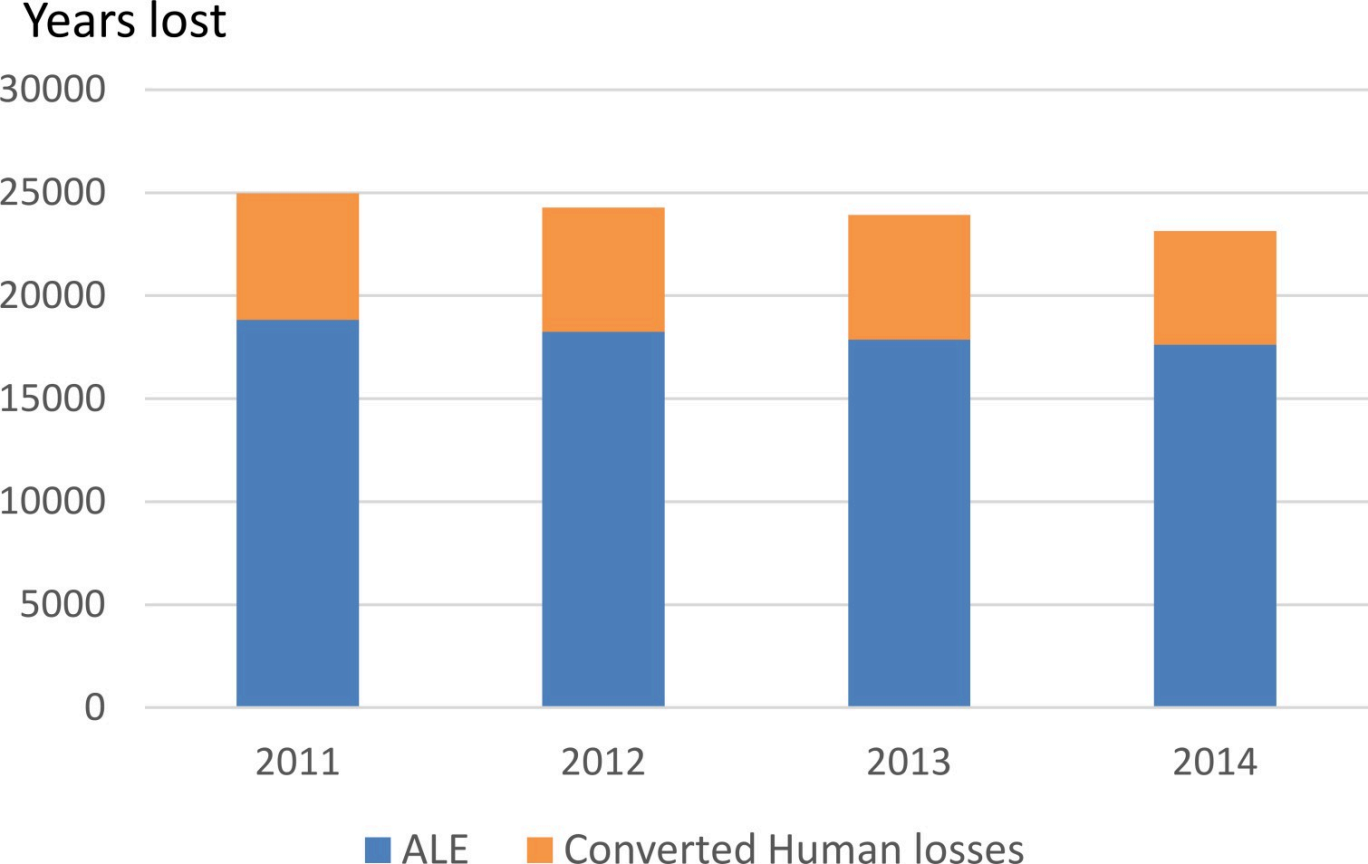
Animal loss equivalents (ALE) and human losses converted to lost years of work.

The total annual economic losses converted in terms of lost years resulted in a mean (min-max) of 24,079 years (23,139–24,972) (i.e. 72 years/100,000 persons) (Fig 10).

## Discussion

This study carried out for the first time an estimation of CE impact on humans and livestock at the national and regional level in Morocco. To better understand CE effects, it is important to estimate its economic impact and the different items that contribute to its burden [39]. However, the accurate assessment is complex, even when estimations are limited to economic and monetary losses [36].

The results of this study indicated that CE caused huge economic losses in Morocco. Specifically, the estimated economic losses (73 million USD per year on average) represented 0.07% of Morocco GDP. Economic losses did not decrease from 2011 to 2014. Similar studies carried out in other countries reported variable economic losses: 14.7 million USD in Tunisia (0.03% of GDP), 200 million USD in Spain (0.01% of GDP), and 89 million USD in Turkey (0.01% of GDP) [27]. Thus, the results for Morocco indicate a higher loss in terms of GDP. Indeed, comparison of total losses among countries is not relevant due to the direct link between GDP and several parameters used for estimations (GNI per capita, value of production), as well as because of contextual and methodological differences. Only comparisons of relative values are therefore relevant (though still affected by possible methodological differences and availability of data), indicating here the particularly high burden caused by CE in Morocco.

Losses of human and animal productivity represented the most important part of CE economic burden in Morocco. This means that most of the huge impact goes unnoticed, explaining its actual neglect. Our study brings information on these unnoticed impacts that may help decision-makers and stimulate efforts towards CE control. Indeed, the major role of human productivity losses in the total economic losses indicates that CE neglect might hamper human development and social justice; because it mainly affects rural populations where physical effort is essential for ensuring livelihood and welfare. Productivity is one of the main drivers of economic growth, and productivity losses can have a significant impact on income [42]. Productivity losses caused by CE also increase the cost of living of the affected families [27]. CE-related animal productivity losses explained most of the monetary losses linked to CE in animals in our study. Similar results were found in other countries [27,34,39,43]. Such losses reflect CE economic impact that is often not known by agricultural managers [27]. The present study, by including human productivity losses in the calculation of the monetary impacts, highlights how CE effects on human and animal productivity have a tremendous impact on rural livelihood and strengthen the need of action by policy makers.

Despite the national control programme launched in 2005, CE-linked economic losses did not significantly decrease in any region. Importantly, in the LBSGE region, where the human incidence of CE is lowest in the country, losses tended to increase during the 2011–2014 period. This could be explained by the poor implementation of the CE control programme in this region. In the MT region that includes also Khénifra, recent studies indeed indicate high CE prevalence in human and animals, and a massive infestation of dogs [5,16]. Therefore, an in-depth analysis of CE prevalence and how the control programme has been implemented is necessary to understand the absence of results. Moreover, to improve the CE national control programme, the economic, ecological, cultural and social specificities of each region must be taken into account.

This study also estimated the DALY and zDALY (i.e. the time lost due to human and animal morbidity and mortality). Quality-adjusted life year (QALY) is another health-adjusted life year metric that could have been used for such estimations. Here, we chose the DALY because it is the most common metric to quantify disease burdens in the Global South. Moreover, it presents the advantage that the addition of livestock losses (ALE) has been proposed to quantify the disease burden in animals in terms of zDALY [17]. The zDALY estimations for CE in other countries were 29.8 years per 100,000 persons per year in Tunisia, 27.2 years per 100,000 persons per year in Iran and 2.86 years per 100,000 persons per year in Spain [17]. Our analysis showed that in Morocco the zDALY was 55 years per 100,000 persons per year, a value nearly double to that found in Tunisia and Iran. Besides infestation rate, this may be explained by differences in livestock size and GNI per capita. Moreover, for our estimation, we did not rely only on CE prevalence at abattoirs, and we estimated a higher prevalence in adult animals, which contributes to the importance of the losses linked to animal production. Estimates of economic losses caused by CE in Iran and Tunisia were based only on CE prevalence at slaughterhouses [21,27]. By applying the same conversion as for ALE to total monetary losses, we expressed in a DALY-like metric the importance of asymptomatic human cases through the loss of productivity, which, as highlighted here above, entails direct consequences in terms of quality of life. These results may directly influence the degree of priority ascribed to a disease by policy makers or the extent to which decision-makers from different sectors may feel concerned or not by the problem.

The multiplicity of components of the present estimation impose important limitations. Indeed, several parameters were subject to uncertainty that we tried to take into account by using Monte Carlo iterations to give a more nuanced image of the calculated impact. Similarly, losses from decreased fertility in camels were not included, due to lack of data. Obviously, the lack of data is linked to the poor surveillance, which in turn may be ascribed to limited awareness of CE importance. Unfortunately, this lack of data further compromises the accurate estimation of CE burden that would be needed to raise an awareness.

Such estimations aiming to fuel public decision-making, the issue of accuracy of estimations may also entail ethical concerns. Indeed, by taking into account different parameters, such as age, gender, environment, and patient employment status, we aimed to produce estimates that are as close as possible to the actual losses. One might observe, however, that the refinements in calculation in human health costs would make poor sense in terms of decision-making, since those refinements appear to affect only slightly the spectrum of values obtained through the Monte Carlo iterations (having highlighted here above the many uncertainties in the parameter estimations). Furthermore, we can point to an ethical issue in the consideration of different values for the illness or death of different citizens of a country according to their gender or employment status. Scaled up at the international level, this issue may be raised in the same way if losses of different countries are to sum. If GNI of each country is used for estimations, human lives across the globe would indeed have various values, to the detriment of the weight of diseases affecting mainly poor countries. Hence, the quest for economic accuracy would deserve some close scrutiny for its practical meaning and ethical value when it comes to valuing health and human life.

Notwithstanding these various considerations, this first estimation, while taking account of the uncertainty around parameters, clearly demonstrates the economic importance of CE nationwide and should encourage efforts in the control and surveillance of the disease in Morocco.

## Conclusion

Using an approach that combines financial and non-financial methods, this study estimated the economic losses caused by CE in humans and livestock in Morocco (nationwide and in the different regions) during the 2011–2014 period. The study showed significant losses in all regions, thus highlighting the poor implementation or lack of effects of the national CE control programme launched in 2005. The losses were mostly unnoticed (i.e. human and animal productivity losses), and this could partly explain why this zoonosis is neglected in Morocco. This study should encourage decision-makers to invest more in CE control and surveillance. Additional studies are now needed to understand the failure of the CE control programme, by analysing the stakeholders’ involvement and the sociological determinants of CE and its control in the various regions of Morocco.

## Supporting information

S1 Table Parameters used to estimate animal organ losses at slaughterhouses. (DOCX)

S2 Table Costs of CE in patients who underwent surgery. (DOCX)

S3 Table Population of Morocco according to the 2014 census (HCP). (DOCX)

S4 Table CE incidence (i.e., individuals undergoing surgery for CE at a public-sector hospital) in the different regions of Morocco. (DOCX)

S5 Table Parameters used to estimate livestock production losses. (DOCX)

S6 Table Age at disease onset (a) and length of hospital stay (data for Khénifra provincial hospital).
(DOCX)

S7 Table Price of different animal products (in Moroccan Dirhams, Dh). (DOCX)

S8 Table Number of ewes and does (those are the words used to define adult females of these two species) per region and per year (per 1000 heads). (DOCX)

S9 Table Number of cows by region and by year (per 1000 heads). (DOCX)
