# Supplementary material for: The socio-economic burden of cystic echinococcosis in Morocco: A combination of estimation method
Source: PLoS Negl Trop Dis. 2020 Jul 31;14(7):e0008410. doi: 10.1371/journal.pntd.0008410 (PMC7423152; doi:10.1371/journal.pntd.0008410)
Supplement: S1 Table — (DOCX) [file pntd.0008410.s001.docx]

Table S1: **Parameters used to estimate animal organ losses at slaughterhouses**. Source: ONSSA

| **Year** | **Region** | **Species** | **Lung condemned (Kg)** | **Liver condemned (Kg)** | **Prevalence of CE**  **(Slaughterhouses)** |
| --- | --- | --- | --- | --- | --- |
| 2011 | CODA | Ovine | 3754 | 2104 | 3.27% |
| 2011 | GC | Ovine | 1733 | 972 | 2.46% |
| 2011 | LBSGE | Ovine | 5608 | 3143 | 8.76% |
| 2011 | MTATA | Ovine | 6004 | 3366 | 8.78% |
| 2011 | MT | Ovine | 4326 | 2425 | 24.95% |
| 2011 | Or | Ovine | 1503 | 842 | 2.89% |
| 2011 | RSZCB | Ovine | 1156 | 648 | 1.86% |
| 2011 | SM | Ovine | 1631 | 915 | 6.90% |
| 2011 | TT | Ovine | 2536 | 1422 | 8.79% |
| 2011 | THTFB | Ovine | 1006 | 564 | 15.19% |
| 2012 | CODA | Ovine | 3355 | 1880 | 4.61% |
| 2012 | GC | Ovine | 2968 | 1664 | 2.13% |
| 2012 | LBSGE | Ovine | 8725 | 4891 | 10.96% |
| 2012 | MTATA | Ovine | 5531 | 3100 | 8.36% |
| 2012 | MT | Ovine | 9583 | 5372 | 21.05% |
| 2012 | Or | Ovine | 9542 | 5348 | 3.03% |
| 2012 | RSZCB | Ovine | 10441 | 5852 | 2.35% |
| 2012 | SM | Ovine | 4337 | 2431 | 6.86% |
| 2012 | TT | Ovine | 11311 | 6340 | 10.07% |
| 2012 | THTFB | Ovine | 9268 | 5195 | 13.05% |
| 2013 | CODA | Ovine | 4855 | 2721 | 4.15% |
| 2013 | GC | Ovine | 1878 | 1052 | 1.25% |
| 2013 | LBSGE | Ovine | 4015 | 2251 | 13.55% |
| 2013 | MTATA | Ovine | 2546 | 1427 | 7.00% |
| 2013 | MT | Ovine | 2752 | 1542 | 20.38% |
| 2013 | Or | Ovine | 1947 | 1092 | 2.29% |
| 2013 | RSZCB | Ovine | 4375 | 2452 | 2.30% |
| 2013 | SM | Ovine | 5540 | 3106 | 6.59% |
| 2013 | TT | Ovine | 4108 | 2302 | 13.81% |
| 2013 | THTFB | Ovine | 1606 | 901 | 16.35% |
| 2014 | CODA | Ovine | 3276 | 1837 | 4.31% |
| 2014 | GC | Ovine | 2815 | 1578 | 1.16% |
| 2014 | LBSGE | Ovine | 3524 | 1975 | 15.61% |
| 2014 | MTATA | Ovine | 1867 | 1046 | 8.11% |
| 2014 | MT | Ovine | 3795 | 2128 | 12.47% |
| 2014 | Or | Ovine | 3494 | 1959 | 2.04% |
| 2014 | RSZCB | Ovine | 6230 | 3492 | 2.10% |
| 2014 | SM | Ovine | 2864 | 1606 | 7.13% |
| 2014 | TT | Ovine | 5310 | 2976 | 16.00% |
| 2014 | THTFB | Ovine | 5298 | 2970 | 15.52% |
| 2011 | CODA | Cattle | 8237 | 5165 | 9.71% |
| 2011 | GC | Cattle | 3822 | 2396 | 13.29% |
| 2011 | LBSGE | Cattle | 14332 | 8988 | 17.42% |
| 2011 | MTATA | Cattle | 17469 | 10955 | 11.33% |
| 2011 | MT | Cattle | 24629 | 15445 | 25.81% |
| 2011 | Or | Cattle | 10143 | 6360 | 8.16% |
| 2011 | RSZCB | Cattle | 14839 | 9305 | 5.24% |
| 2011 | SM | Cattle | 30640 | 19214 | 15.61% |
| 2011 | TT | Cattle | 2514 | 1576 | 10.68% |
| 2011 | THTFB | Cattle | 1664 | 1044 | 21.14% |
| 2012 | CODA | Cattle | 3347 | 2099 | 6.17% |
| 2012 | GC | Cattle | 5841 | 3663 | 8.42% |
| 2012 | LBSGE | Cattle | 8725 | 5472 | 19.91% |
| 2012 | MTATA | Cattle | 7616 | 4775 | 10.50% |
| 2012 | MT | Cattle | 20917 | 13117 | 28.28% |
| 2012 | Or | Cattle | 21620 | 13557 | 6.53% |
| 2012 | RSZCB | Cattle | 13392 | 8398 | 6.96% |
| 2012 | SM | Cattle | 6341 | 3976 | 11.22% |
| 2012 | TT | Cattle | 23553 | 14770 | 12.87% |
| 2012 | THTFB | Cattle | 26275 | 16477 | 15.19% |
| 2013 | CODA | Cattle | 3928 | 2463 | 5.69% |
| 2013 | GC | Cattle | 1375 | 862 | 7.50% |
| 2013 | LBSGE | Cattle | 3617 | 2268 | 25.65% |
| 2013 | MTATA | Cattle | 3492 | 2190 | 9.27% |
| 2013 | MT | Cattle | 15077 | 9455 | 29.66% |
| 2013 | Or | Cattle | 8266 | 5183 | 4.69% |
| 2013 | RSZCB | Cattle | 19503 | 12230 | 6.15% |
| 2013 | SM | Cattle | 23204 | 14550 | 10.04% |
| 2013 | TT | Cattle | 14328 | 8985 | 15.06% |
| 2013 | THTFB | Cattle | 5474 | 3433 | 17.21% |
| 2014 | CODA | Cattle | 14463 | 9070 | 5.85% |
| 2014 | GC | Cattle | 17130 | 10742 | 8.50% |
| 2014 | LBSGE | Cattle | 9103 | 5709 | 31.55% |
| 2014 | MTATA | Cattle | 4354 | 2731 | 2.29% |
| 2014 | MT | Cattle | 12257 | 7686 | 21.04% |
| 2014 | Or | Cattle | 11712 | 7344 | 4.28% |
| 2014 | RSZCB | Cattle | 13944 | 8744 | 5.72% |
| 2014 | SM | Cattle | 5765 | 3615 | 8.98% |
| 2014 | TT | Cattle | 19317 | 12113 | 14.51% |
| 2014 | THTFB | Cattle | 20762 | 13019 | 14.64% |
| 2011 | CODA | Goat | 273 | 136 | 1.64% |
| 2011 | GC | Goat | 47 | 23 | 2.40% |
| 2011 | LBSGE | Goat | 407 | 203 | 1.70% |
| 2011 | MTATA | Goat | 543 | 271 | 4.85% |
| 2011 | MT | Goat | 9 | 4 | 12.70% |
| 2011 | Or | Goat | 39 | 20 | 1.92% |
| 2011 | RSZCB | Goat | 9 | 5 | 1.58% |
| 2011 | SM | Goat | 43 | 22 | 1.76% |
| 2011 | TT | Goat | 1279 | 639 | 6.03% |
| 2011 | THTFB | Goat | 1065 | 532 | 8.73% |
| 2012 | CODA | Goat | 2917 | 1458 | 0.51% |
| 2012 | GC | Goat | 4031 | 2015 | 3.56% |
| 2012 | LBSGE | Goat | 2286 | 1143 | 4.03% |
| 2012 | MTATA | Goat | 853 | 426 | 3.23% |
| 2012 | MT | Goat | 2705 | 1352 | 10.62% |
| 2012 | Or | Goat | 2638 | 1319 | 2.52% |
| 2012 | RSZCB | Goat | 3298 | 1648 | 1.58% |
| 2012 | SM | Goat | 583 | 291 | 1.92% |
| 2012 | TT | Goat | 3766 | 1883 | 6.69% |
| 2012 | THTFB | Goat | 2618 | 1309 | 7.07% |
| 2013 | CODA | Goat | 844 | 422 | 0.88% |
| 2013 | GC | Goat | 489 | 244 | 1.79% |
| 2013 | LBSGE | Goat | 1088 | 544 | 6.19% |
| 2013 | MTATA | Goat | 801 | 400 | 4.16% |
| 2013 | MT | Goat | 485 | 242 | 12.85% |
| 2013 | Or | Goat | 295 | 147 | 2.15% |
| 2013 | RSZCB | Goat | 784 | 392 | 1.57% |
| 2013 | SM | Goat | 992 | 496 | 1.23% |
| 2013 | TT | Goat | 3142 | 1571 | 5.85% |
| 2013 | THTFB | Goat | 1600 | 800 | 8.60% |
| 2014 | CODA | Goat | 1988 | 994 | 1.56% |
| 2014 | GC | Goat | 1390 | 695 | 2.34% |
| 2014 | LBSGE | Goat | 1552 | 776 | 9.10% |
| 2014 | MTATA | Goat | 698 | 349 | 5.97% |
| 2014 | MT | Goat | 1448 | 724 | 8.15% |
| 2014 | Or | Goat | 1325 | 663 | 2.20% |
| 2014 | RSZCB | Goat | 1665 | 832 | 1.47% |
| 2014 | SM | Goat | 623 | 311 | 1.02% |
| 2014 | TT | Goat | 1366 | 683 | 7.34% |
| 2014 | THTFB | Goat | 1403 | 701 | 8.09% |
| 2011 | CODA | Camel | 0 | 0 | 0% |
| 2011 | GC | Camel | 6 | 2 | 80.00% |
| 2011 | LBSGE | Camel | 3431 | 1025 | 9.82% |
| 2011 | MTATA | Camel | 22 | 6 | 4.02% |
| 2011 | MT | Camel | 1 | 0 | 2.17% |
| 2011 | Or | Camel | 0 | 0 | 0% |
| 2011 | RSZCB | Camel | 30 | 9 | 2.05% |
| 2011 | SM | Camel | 118 | 35 | 6.55% |
| 2011 | TT | Camel | 0 | 0 | 0% |
| 2011 | THTFB | Camel | 56 | 17 | 36.23% |
| 2012 | CODA | Camel | 0 | 0 | 0% |
| 2012 | GC | Camel | 0 | 0 | 8.42% |
| 2012 | LBSGE | Camel | 3308 | 988 | 25.03% |
| 2012 | MTATA | Camel | 8 | 3 | 5.90% |
| 2012 | MT | Camel | 5 | 1 | 3.08% |
| 2012 | Or | Camel | 0 | 0 | 0.38% |
| 2012 | RSZCB | Camel | 42 | 13 | 5.03% |
| 2012 | SM | Camel | 49 | 15 | 4.24% |
| 2012 | TT | Camel | 0 | 0 | 0.00% |
| 2012 | THTFB | Camel | 20 | 6 | 20.75% |
| 2013 | CODA | Camel | 0 | 0 | 0% |
| 2013 | GC | Camel | 0 | 0 | 0% |
| 2013 | LBSGE | Camel | 5874 | 1754 | 28.82% |
| 2013 | MTATA | Camel | 15 | 5 | 4.80% |
| 2013 | MT | Camel | 0 | 0 | 0% |
| 2013 | Or | Camel | 59 | 17 | 5.95% |
| 2013 | RSZCB | Camel | 39 | 12 | 2.84% |
| 2013 | SM | Camel | 42 | 12 | 2.05% |
| 2013 | TT | Camel | 0 | 0 | 0% |
| 2013 | THTFB | Camel | 16 | 5 | 2.94% |
| 2014 | CODA | Camel | 28 | 9 | 4.99% |
| 2014 | GC | Camel | 0 | 0 | 0% |
| 2014 | LBSGE | Camel | 4069 | 1216 | 23.49% |
| 2014 | MTATA | Camel | 329 | 98 | 27.98% |
| 2014 | MT | Camel | 0 | 0 | 0% |
| 2014 | Or | Camel | 0 | 0 | 0% |
| 2014 | RSZCB | Camel | 55 | 17 | 4% |
| 2014 | SM | Camel | 12 | 4 | 0.68% |
| 2014 | TT | Camel | 0 | 0 | 0% |
| 2014 | THTFB | Camel | 17 | 5 | 7.81% |
