# Supplementary material for: The socio-economic burden of cystic echinococcosis in Morocco: A combination of estimation method
Source: PLoS Negl Trop Dis. 2020 Jul 31;14(7):e0008410. doi: 10.1371/journal.pntd.0008410 (PMC7423152; doi:10.1371/journal.pntd.0008410)
Supplement: S2 Table — (DOCX) [file pntd.0008410.s002.docx]

Table S2: Costs of CE in patients who underwent surgery (in Moroccan Dirhams DH). Source: Khenifra Hospital

| **Expense items** | **Cost per patient (DH)** | |
| --- | --- | --- |
|  | **Min** | **Max** |
| Consultations | 300 | 400 |
| Medical imaging | 200 | 400 |
| Pharmaceuticals | 200 | 350 |
| Laboratory analysis | 200 | 350 |
| Cost of abdominal surgery | 1500 | 2900 |
| Cost of thoracic surgery | 8000 | 12000 |
| Postoperative treatment | 400 | 500 |
| Night cost of hospitalization | 80 | 100 |
| Accompaniment of a family member | 700 | 800 |
| Transport | 300 | 600 |
| Extra hospital food | 200 | 500 |
| Cost of invalidity of patients before surgery | 1000 | 1500 |
| Complications | 800 | 1400 |
| Risk of recurrence | 200 | 300 |
