# Supplementary material for: The socio-economic burden of cystic echinococcosis in Morocco: A combination of estimation method
Source: PLoS Negl Trop Dis. 2020 Jul 31;14(7):e0008410. doi: 10.1371/journal.pntd.0008410 (PMC7423152; doi:10.1371/journal.pntd.0008410)
Supplement: S3 Table — (DOCX) [file pntd.0008410.s003.docx]

Table S3: Population of Morocco according to the 2014 census (HCP)

| **Region** | **Region (code)** | **Population** |
| --- | --- | --- |
| Chaouia Ouardigha Doukkala Abda | CODA | 4,077,040 |
| Grand Casablanca | GC | 4,268,105 |
| Laâyoune Boujdour Sakia El Hamra Guelmim Essmara | LBSGE | 944,470 |
| Marrakech Tensift Al Haouz Tadla Azilal | MTATA | 5,184,149 |
| Meknes Tafilalet | MT | 2,316,865 |
| Oriental | Or | 2,314,346 |
| Rabat Salé Zemmour Zaer Chrarda Bni Hssen | RSZCB | 4,580,866 |
| Souss Massa Draâ | SM | 2,676,847 |
| Tanger Tetouan | TT | 3,107,723 |
| Taza Al Hoceima Taounate Fes Boulemane | THTFB | 3,615,331 |
