# Supplementary material for: The socio-economic burden of cystic echinococcosis in Morocco: A combination of estimation method
Source: PLoS Negl Trop Dis. 2020 Jul 31;14(7):e0008410. doi: 10.1371/journal.pntd.0008410 (PMC7423152; doi:10.1371/journal.pntd.0008410)
Supplement: S4 Table — (DOCX) [file pntd.0008410.s004.docx]

Table S4: CE incidence (i.e., individuals undergoing surgery for CE at a public-sector hospital) in the different regions of Morocco

| **Region** | **Region (code)** | **2011** | **2012** | **2013** | **2014** |
| --- | --- | --- | --- | --- | --- |
| Chaouia Ouardigha Doukkala Abda | CODA | 291 | 155 | 103 | 105 |
| Grand Casablanca | GC | 63 | 121 | 123 | 5 |
| Laâyoune Boujdour Sakia El Hamra Guelmim Essmara | LBSGE | 23 | 13 | 16 | 11 |
| Marrakech Tensift Al Haouz Tadla Azilal | MTATA | 231 | 142 | 116 | 143 |
| Meknes Tafilalet | MT | 183 | 166 | 159 | 119 |
| Oriental | Or | 81 | 55 | 42 | 38 |
| Rabat Salé Zemmour Zaer Chrarda Bni Hssen | RSZCB | 223 | 188 | 131 | 112 |
| Souss Massa Draâ | SM | 90 | 75 | 69 | 75 |
| Tanger Tetouan | TT | 106 | 87 | 46 | 39 |
| Taza Al Hoceima Taounate Fes Boulemane | THTFB | 175 | 80 | 128 | 75 |
