# Supplementary material for: The socio-economic burden of cystic echinococcosis in Morocco: A combination of estimation method
Source: PLoS Negl Trop Dis. 2020 Jul 31;14(7):e0008410. doi: 10.1371/journal.pntd.0008410 (PMC7423152; doi:10.1371/journal.pntd.0008410)
Supplement: S5 Table — (DOCX) [file pntd.0008410.s005.docx]

Table S5: Parameters used to estimate livestock production losses

| **Year** | **Region** | **Species** | **Average milk per cow (Kg)** | **Slaughtered animals affected** | **Weight by carcass (kg)** | **Female reproductive** | **Production of wood by animal (kg)** |
| --- | --- | --- | --- | --- | --- | --- | --- |
| 2011 | CODA | Ovine | - | 7678 | 65 | 671172 | 2.6 |
| 2011 | GC | Ovine | - | 7056 | 65 | 1248357 | 2.6 |
| 2011 | LBSGE | Ovine | - | 4645 | 65 | 79719 | 2.6 |
| 2011 | MTATA | Ovine | - | 41009 | 65 | 1542221 | 2.6 |
| 2011 | MT | Ovine | - | 23982 | 65 | 1236655 | 2.6 |
| 2011 | Or | Ovine | - | 11774 | 65 | 1050307 | 2.6 |
| 2011 | RSZCB | Ovine | - | 4271 | 65 | 847036 | 2.6 |
| 2011 | SM | Ovine | - | 10655 | 65 | 451354 | 2.6 |
| 2011 | TT | Ovine | - | 8280 | 65 | 477050 | 2.6 |
| 2011 | THTFB | Ovine | - | 25234 | 65 | 1429466 | 2.6 |
| 2012 | CODA | Ovine | - | 11444 | 59 | 687244 | 2.6 |
| 2012 | GC | Ovine | - | 4347 | 59 | 1264948 | 2.6 |
| 2012 | LBSGE | Ovine | - | 5509 | 59 | 100114 | 2.6 |
| 2012 | MTATA | Ovine | - | 41218 | 59 | 1595322 | 2.6 |
| 2012 | MT | Ovine | - | 21133 | 59 | 1182093 | 2.6 |
| 2012 | Or | Ovine | - | 13646 | 59 | 1165972 | 2.6 |
| 2012 | RSZCB | Ovine | - | 7707 | 59 | 802747 | 2.6 |
| 2012 | SM | Ovine | - | 9112 | 59 | 560883 | 2.6 |
| 2012 | TT | Ovine | - | 8940 | 59 | 476818 | 2.6 |
| 2012 | THTFB | Ovine | - | 17049 | 59 | 1374431 | 2.6 |
| 2013 | CODA | Ovine | - | 9387 | 58 | 692535 | 2.6 |
| 2013 | GC | Ovine | - | 1794 | 58 | 1222737 | 2.5 |
| 2013 | LBSGE | Ovine | - | 6889 | 58 | 111131 | 2.5 |
| 2013 | MTATA | Ovine | - | 37165 | 58 | 1551490 | 2.6 |
| 2013 | MT | Ovine | - | 22372 | 58 | 1213172 | 2.5 |
| 2013 | Or | Ovine | - | 9698 | 58 | 1267997 | 2.7 |
| 2013 | RSZCB | Ovine | - | 5407 | 58 | 757769 | 2.6 |
| 2013 | SM | Ovine | - | 8319 | 58 | 574366 | 2.6 |
| 2013 | TT | Ovine | - | 11634 | 58 | 513114 | 2.6 |
| 2013 | THTFB | Ovine | - | 21519 | 58 | 1356735 | 2.7 |
| 2014 | CODA | Ovine | - | 9260 | 68 | 641124 | 2.6 |
| 2014 | GC | Ovine | - | 2919 | 68 | 1178771 | 2.7 |
| 2014 | LBSGE | Ovine | - | 7880 | 68 | 117785 | 2.7 |
| 2014 | MTATA | Ovine | - | 33797 | 68 | 1623021 | 2.6 |
| 2014 | MT | Ovine | - | 16722 | 68 | 1134411 | 2.7 |
| 2014 | Or | Ovine | - | 6346 | 68 | 1199702 | 2.5 |
| 2014 | RSZCB | Ovine | - | 6371 | 68 | 766487 | 2.6 |
| 2014 | SM | Ovine | - | 7377 | 68 | 568560 | 2.6 |
| 2014 | TT | Ovine | - | 11829 | 68 | 511141 | 2.7 |
| 2014 | THTFB | Ovine | - | 18406 | 68 | 1401359 | 2.6 |
| 2011 | CODA | Cattle | 1802 | 9208 | 314 | 145187 | - |
| 2011 | GC | Cattle | 1802 | 12738 | 314 | 48619 | - |
| 2011 | LBSGE | Cattle | 1802 | 1110 | 314 | 144479 | - |
| 2011 | MTATA | Cattle | 1802 | 14661 | 314 | 221448 | - |
| 2011 | MT | Cattle | 1802 | 6537 | 314 | 619 | - |
| 2011 | Or | Cattle | 1802 | 2601 | 314 | 198984 | - |
| 2011 | RSZCB | Cattle | 1802 | 5107 | 314 | 33820 | - |
| 2011 | SM | Cattle | 1802 | 10342 | 314 | 200913 | - |
| 2011 | TT | Cattle | 1802 | 3880 | 314 | 86640 | - |
| 2011 | THTFB | Cattle | 1802 | 13781 | 314 | 196794 | - |
| 2012 | CODA | Cattle | 2008 | 7243 | 335 | 158042 | - |
| 2012 | GC | Cattle | 2008 | 7341 | 335 | 46212 | - |
| 2012 | LBSGE | Cattle | 2008 | 1751 | 335 | 141914 | - |
| 2012 | MTATA | Cattle | 2008 | 14511 | 335 | 235227 | - |
| 2012 | MT | Cattle | 2008 | 7972 | 335 | 810 | - |
| 2012 | Or | Cattle | 2008 | 2638 | 335 | 197006 | - |
| 2012 | RSZCB | Cattle | 2008 | 6380 | 335 | 32210 | - |
| 2012 | SM | Cattle | 2008 | 8725 | 335 | 196653 | - |
| 2012 | TT | Cattle | 2008 | 4926 | 335 | 89308 | - |
| 2012 | THTFB | Cattle | 2008 | 8554 | 335 | 200873 | - |
| 2013 | CODA | Cattle | 1879 | 8623 | 317 | 167681 | - |
| 2013 | GC | Cattle | 1879 | 4793 | 317 | 46300 | - |
| 2013 | LBSGE | Cattle | 1879 | 2383 | 317 | 148640 | - |
| 2013 | MTATA | Cattle | 1879 | 16280 | 317 | 234051 | - |
| 2013 | MT | Cattle | 1879 | 14152 | 317 | 789 | - |
| 2013 | Or | Cattle | 1879 | 2234 | 317 | 173832 | - |
| 2013 | RSZCB | Cattle | 1879 | 6119 | 317 | 32946 | - |
| 2013 | SM | Cattle | 1879 | 8800 | 317 | 201056 | - |
| 2013 | TT | Cattle | 1879 | 6644 | 317 | 89071 | - |
| 2013 | THTFB | Cattle | 1879 | 13510 | 317 | 187711 | - |
| 2014 | CODA | Cattle | 2103 | 10032 | 272 | 149401 | - |
| 2014 | GC | Cattle | 2103 | 10114 | 272 | 43369 | - |
| 2014 | LBSGE | Cattle | 2103 | 3948 | 272 | 144348 | - |
| 2014 | MTATA | Cattle | 2103 | 16117 | 272 | 242990 | - |
| 2014 | MT | Cattle | 2103 | 13449 | 272 | 683 | - |
| 2014 | Or | Cattle | 2103 | 2009 | 272 | 192028 | - |
| 2014 | RSZCB | Cattle | 2103 | 7877 | 272 | 33819 | - |
| 2014 | SM | Cattle | 2103 | 8994 | 272 | 207159 | - |
| 2014 | TT | Cattle | 2103 | 6307 | 272 | 91577 | - |
| 2014 | THTFB | Cattle | 2103 | 11604 | 272 | 189156 | - |
| 2011 | CODA | Goat | - | 919 | 18 | 403294 | - |
| 2011 | GC | Goat | - | 50 | 18 | 411715 | - |
| 2011 | LBSGE | Goat | - | 3582 | 18 | 345911 | - |
| 2011 | MTATA | Goat | - | 14013 | 18 | 22468 | - |
| 2011 | MT | Goat | - | 8670 | 18 | 91292 | - |
| 2011 | Or | Goat | - | 2584 | 18 | 500422 | - |
| 2011 | RSZCB | Goat | - | 817 | 18 | 146304 | - |
| 2011 | SM | Goat | - | 7897 | 18 | 94068 | - |
| 2011 | TT | Goat | - | 3981 | 18 | 418812 | - |
| 2011 | THTFB | Goat | - | 7567 | 18 | 447484 | - |
| 2012 | CODA | Goat | - | 253 | 24 | 437072 | - |
| 2012 | GC | Goat | - | 205 | 24 | 405417 | - |
| 2012 | LBSGE | Goat | - | 4701 | 24 | 346019 | - |
| 2012 | MTATA | Goat | - | 9104 | 24 | 22411 | - |
| 2012 | MT | Goat | - | 5941 | 24 | 94186 | - |
| 2012 | Or | Goat | - | 3733 | 24 | 525208 | - |
| 2012 | RSZCB | Goat | - | 1134 | 24 | 143556 | - |
| 2012 | SM | Goat | - | 7626 | 24 | 143556 | - |
| 2012 | TT | Goat | - | 4107 | 24 | 454749 | - |
| 2012 | THTFB | Goat | - | 5093 | 24 | 427558 | - |
| 2013 | CODA | Goat | - | 548 | 21 | 473856 | - |
| 2013 | GC | Goat | - | 22 | 21 | 371278 | - |
| 2013 | LBSGE | Goat | - | 7847 | 21 | 337534 | - |
| 2013 | MTATA | Goat | - | 12297 | 21 | 30708 | - |
| 2013 | MT | Goat | - | 10238 | 21 | 100279 | - |
| 2013 | Or | Goat | - | 2861 | 21 | 409996 | - |
| 2013 | RSZCB | Goat | - | 960 | 21 | 152538 | - |
| 2013 | SM | Goat | - | 5096 | 21 | 63984 | - |
| 2013 | TT | Goat | - | 4334 | 21 | 530067 | - |
| 2013 | THTFB | Goat | - | 5885 | 21 | 436384 | - |
| 2014 | CODA | Goat | - | 863 | 27 | 436438 | - |
| 2014 | GC | Goat | - | 80 | 27 | 384869 | - |
| 2014 | LBSGE | Goat | - | 9442 | 27 | 329089 | - |
| 2014 | MTATA | Goat | - | 14535 | 27 | 32347 | - |
| 2014 | MT | Goat | - | 6981 | 27 | 95080 | - |
| 2014 | Or | Goat | - | 2182 | 27 | 513728 | - |
| 2014 | RSZCB | Goat | - | 1132 | 27 | 155245 | - |
| 2014 | SM | Goat | - | 3248 | 27 | 80991 | - |
| 2014 | TT | Goat | - | 4320 | 27 | 481750 | - |
| 2014 | THTFB | Goat | - | 5614 | 27 | 440952 | - |
| 2011 | CODA | Camel | - | 0 | 420 | - | - |
| 2011 | GC | Camel | - | 56 | 420 | - | - |
| 2011 | LBSGE | Camel | - | 1567 | 420 | - | - |
| 2011 | MTATA | Camel | - | 84 | 420 | - | - |
| 2011 | MT | Camel | - | 1 | 420 | - | - |
| 2011 | Or | Camel | - | 0 | 420 | - | - |
| 2011 | RSZCB | Camel | - | 9 | 420 | - | - |
| 2011 | SM | Camel | - | 88 | 420 | - | - |
| 2011 | TT | Camel | - | 0 | 420 | - | - |
| 2011 | THTFB | Camel | - | 25 | 420 | - | - |
| 2012 | CODA | Camel | - | 0 | 420 | - | - |
| 2012 | GC | Camel | - | 0 | 420 | - | - |
| 2012 | LBSGE | Camel | - | 3623 | 420 | - | - |
| 2012 | MTATA | Camel | - | 20 | 420 | - | - |
| 2012 | MT | Camel | - | 2 | 420 | - | - |
| 2012 | Or | Camel | - | 1 | 420 | - | - |
| 2012 | RSZCB | Camel | - | 18 | 420 | - | - |
| 2012 | SM | Camel | - | 50 | 420 | - | - |
| 2012 | TT | Camel | - | 0 | 420 | - | - |
| 2012 | THTFB | Camel | - | 11 | 420 | - | - |
| 2013 | CODA | Camel | - | 0 | 420 | - | - |
| 2013 | GC | Camel | - | 0 | 420 | - | - |
| 2013 | LBSGE | Camel | - | 4373 | 420 | - | - |
| 2013 | MTATA | Camel | - | 18 | 420 | - | - |
| 2013 | MT | Camel | - | 0 | 420 | - | - |
| 2013 | Or | Camel | - | 84 | 420 | - | - |
| 2013 | RSZCB | Camel | - | 8 | 420 | - | - |
| 2013 | SM | Camel | - | 29 | 420 | - | - |
| 2013 | TT | Camel | - | 0 | 420 | - | - |
| 2013 | THTFB | Camel | - | 2 | 420 | - | - |
| 2014 | CODA | Camel | - | 55 | 420 | - | - |
| 2014 | GC | Camel | - | 0 | 420 | - | - |
| 2014 | LBSGE | Camel | - | 3087 | 420 | - | - |
| 2014 | MTATA | Camel | - | 183 | 420 | - | - |
| 2014 | MT | Camel | - | 0 | 420 | - | - |
| 2014 | Or | Camel | - | 0 | 420 | - | - |
| 2014 | RSZCB | Camel | - | 11 | 420 | - | - |
| 2014 | SM | Camel | - | 18 | 420 | - | - |
| 2014 | TT | Camel | - | 0 | 420 | - | - |
| 2014 | THTFB | Camel | - | 5 | 420 | - | - |
