# Supplementary material for: The socio-economic burden of cystic echinococcosis in Morocco: A combination of estimation method
Source: PLoS Negl Trop Dis. 2020 Jul 31;14(7):e0008410. doi: 10.1371/journal.pntd.0008410 (PMC7423152; doi:10.1371/journal.pntd.0008410)
Supplement: S6 Table — Age at disease onset (a) and length of hospital stay (data for Khénifra provincial hospital). (DOCX) [file pntd.0008410.s006.docx]

Table S6: Age at disease onset (a) and length of hospital stay (data for Khénifra provincial hospital). “a” corresponds to the patient's age at the time of the operation.

| **Year** | **Sex** | **Age range** | **Age at disease onset (year)** | **length of hospital stay (days)** |
| --- | --- | --- | --- | --- |
| 2011 | Women | 15-19 | 16 | 16 |
| 2011 | Women | 20-24 | 22 | 7 |
| 2011 | Women | 25-29 | 29 | 15 |
| 2011 | Women | 30-34 | 30 | 4 |
| 2011 | Women | 30-34 | 31 | 11 |
| 2011 | Women | 40-44 | 41 | 3 |
| 2011 | Women | 40-44 | 42 | 16 |
| 2011 | Women | 40-44 | 42 | 7 |
| 2011 | Women | 40-44 | 44 | 5 |
| 2011 | Women | 45-49 | 45 | 3 |
| 2011 | Women | 50-54 | 50 | 10 |
| 2011 | Women | 50-54 | 53 | 11 |
| 2011 | Women | 55-59 | 55 | 17 |
| 2011 | Women | 55-59 | 56 | 16 |
| 2011 | Women | 70-74 | 71 | 13 |
| 2011 | Men | 15-19 | 17 | 10 |
| 2011 | Men | 20-24 | 20 | 13 |
| 2011 | Men | 20-24 | 21 | 17 |
| 2011 | Men | 30-34 | 31 | 5 |
| 2011 | Men | 30-34 | 32 | 8 |
| 2011 | Men | 30-34 | 33 | 12 |
| 2011 | Men | 45-49 | 47 | 20 |
| 2011 | Men | 55-59 | 56 | 13 |
| 2011 | Men | 65-69 | 65 | 10 |
| 2011 | Men | 65-69 | 66 | 17 |
| 2012 | Women | 20-24 | 20 | 13 |
| 2012 | Women | 25-29 | 26 | 12 |
| 2012 | Women | 25-29 | 26 | 7 |
| 2012 | Women | 25-29 | 28 | 7 |
| 2012 | Women | 30-34 | 30 | 4 |
| 2012 | Women | 30-34 | 31 | 10 |
| 2012 | Women | 30-34 | 31 | 3 |
| 2012 | Women | 40-44 | 41 | 6 |
| 2012 | Women | 40-44 | 42 | 7 |
| 2012 | Women | 40-44 | 43 | 10 |
| 2012 | Women | 40-44 | 48 | 14 |
| 2012 | Men | 20-24 | 20 | 9 |
| 2012 | Men | 25-29 | 26 | 20 |
| 2012 | Men | 40-44 | 40 | 10 |
| 2012 | Men | 40-44 | 40 | 4 |
| 2012 | Men | 40-44 | 43 | 16 |
| 2012 | Men | 50-54 | 53 | 3 |
| 2013 | Women | 25-29 | 27 | 4 |
| 2013 | Women | 30-34 | 30 | 6 |
| 2013 | Women | 30-34 | 32 | 16 |
| 2013 | Women | 35-39 | 35 | 7 |
| 2013 | Women | 40-44 | 41 | 3 |
| 2013 | Women | 40-44 | 43 | 6 |
| 2013 | Women | 50-54 | 53 | 10 |
| 2013 | Women | 50-54 | 54 | 3 |
| 2013 | Women | 60-64 | 60 | 10 |
| 2013 | Women | 60-64 | 60 | 4 |
| 2013 | Women | 60-64 | 64 | 14 |
| 2013 | Men | 30-34 | 30 | 8 |
| 2013 | Men | 30-34 | 31 | 6 |
| 2013 | Men | 30-34 | 33 | 5 |
| 2013 | Men | 30-34 | 33 | 6 |
| 2013 | Men | 35-39 | 38 | 5 |
| 2013 | Men | 35-39 | 39 | 3 |
| 2013 | Men | 40-44 | 43 | 3 |
| 2013 | Men | 50-54 | 50 | 4 |
| 2013 | Men | 50-54 | 50 | 3 |
| 2013 | Men | 50-54 | 50 | 5 |
| 2013 | Men | 55-59 | 59 | 3 |
| 2013 | Men | 60-64 | 60 | 3 |
| 2013 | Men | 60-64 | 60 | 5 |
| 2013 | Men | 60-64 | 63 | 9 |
| 2013 | Men | 70-74 | 70 | 6 |
| 2013 | Men | 70-74 | 70 | 7 |
| 2014 | Women | 30-34 | 30 | 3 |
| 2014 | Women | 30-34 | 32 | 5 |
| 2014 | Women | 35-39 | 35 | 5 |
| 2014 | Women | 35-39 | 36 | 5 |
| 2014 | Women | 35-39 | 38 | 2 |
| 2014 | Women | 35-39 | 39 | 9 |
| 2014 | Women | 40-44 | 40 | 5 |
| 2014 | Women | 40-44 | 40 | 13 |
| 2014 | Women | 40-44 | 40 | 3 |
| 2014 | Women | 40-44 | 41 | 3 |
| 2014 | Women | 40-44 | 43 | 6 |
| 2014 | Women | 45-49 | 45 | 3 |
| 2014 | Women | 45-49 | 46 | 3 |
| 2014 | Women | 45-49 | 48 | 8 |
| 2014 | Women | 50-54 | 50 | 4 |
| 2014 | Women | 50-54 | 50 | 6 |
| 2014 | Women | 50-54 | 52 | 8 |
| 2014 | Women | 55-59 | 56 | 3 |
| 2014 | Women | 60-64 | 60 | 5 |
| 2014 | Women | 60-64 | 60 | 34 |
| 2014 | Women | 60-64 | 61 | 10 |
| 2014 | Men | 20-24 | 23 | 7 |
| 2014 | Men | 35-39 | 39 | 7 |
| 2014 | Men | 40-44 | 40 | 10 |
| 2014 | Men | 40-44 | 40 | 7 |
| 2014 | Men | 40-44 | 41 | 6 |
| 2014 | Men | 45-49 | 45 | 1 |
| 2014 | Men | 50-54 | 51 | 7 |
| 2014 | Men | 50-54 | 52 | 9 |
| 2014 | Men | 55-59 | 58 | 11 |
| 2014 | Men | 60-64 | 62 | 9 |
