# Supplementary material for: The socio-economic burden of cystic echinococcosis in Morocco: A combination of estimation method
Source: PLoS Negl Trop Dis. 2020 Jul 31;14(7):e0008410. doi: 10.1371/journal.pntd.0008410 (PMC7423152; doi:10.1371/journal.pntd.0008410)
Supplement: S7 Table — (DOCX) [file pntd.0008410.s007.docx]

Table S7: Price of different animal products (in Moroccan Dirhams, Dh). Official exchange rate (LCU per US$, period average), USD to Dh: 2011: 8.09; 2012: 8.63; 2013: 8.41 and 2014: 8.41.

|  | **Price (Dh) (Min)** | **Price (Dh) (Max)** |
| --- | --- | --- |
| **Lungs (kg)** | 25 | 30 |
| **Liver (kg)** | 70 | 100 |
| **Price 1 liter of milk (dh)** | 3.4 | 4.8 |
| **Meat price (Dh) (sheep, goat, cattle)** | 50 | 70 |
| **Meat price (Dh) (camel)** | 60 | 70 |
| **Price of newborn (dh) (lamb)** | 300 | 700 |
| **Price of newborn (dh) (veal)** | 1500 | 3000 |
| **Price of newborn (dh) (young goat)** | 250 | 500 |
| **Price of 1kg wool (dh)** | 1.5 | 2 |
