# Supplementary material for: The socio-economic burden of cystic echinococcosis in Morocco: A combination of estimation method
Source: PLoS Negl Trop Dis. 2020 Jul 31;14(7):e0008410. doi: 10.1371/journal.pntd.0008410 (PMC7423152; doi:10.1371/journal.pntd.0008410)
Supplement: S8 Table — (DOCX) [file pntd.0008410.s008.docx]

Table S8: Number of ewes and does (those are the words used to define adult females of these two species) per region and per year (per 1000 heads). Source HCP

|  |  |  | 1 à <2 years old | 2 à <6  years old | +6  years old | 6 à 12 month | <6  month |
| --- | --- | --- | --- | --- | --- | --- | --- |
| 2011 | **Meknes Tafilalet** | Goat | 96.1801853 | 307.114283 | 110.080142 | 76.214761 | 102.56537 |
| 2011 | **Chaouia Ouardigha Doukkala Abda** | Goat | 149.128004 | 262.5872 | 8.4018895 | 100.492028 | 76.1431375 |
| 2011 | **Taza Alhoceima Taounate Fes Boulemane** | Goat | 70.9244751 | 274.986909 | 23.2777601 | 53.5274326 | 57.8420415 |
| 2011 | **Grand Casablanca** | Goat | 4.22106712 | 18.2467328 | 8.548349 | 2.51407264 | 5.63416277 |
| 2011 | **Laayoune Boujdour Sakia El hamra Guelmim Essmara** | Goat | 32.9601893 | 58.3321935 | 0 | 12.0331517 | 13.0201466 |
| 2011 | **Marrakech Tensift Al Haouz Tadla Azilal** | Goat | 107.875498 | 392.546021 | 132.050398 | 92.5099695 | 138.990855 |
| 2011 | **Oriental** | Goat | 28.8329192 | 117.471095 | 9.52416672 | 12.4313154 | 31.92582 |
| 2011 | **Rabat Sale zemmour Zaer Chrarda Bni Hssen** | Goat | 20.1548667 | 73.9128162 | 8.0705768 | 14.0708354 | 13.9219667 |
| 2011 | **Souss Massa Draâ** | Goat | 160.368711 | 258.44312 | 35.4625249 | 86.0584241 | 51.3745278 |
| 2011 | **Tanger Tetouan** | Goat | 97.3942722 | 350.089879 | 45.0899064 | 100.035458 | 78.3413306 |
| 2012 | **Meknes Tafilalet** | Goat | 116.709357 | 320.362208 | 140.689764 | 88.5835483 | 119.29465 |
| 2012 | **Chaouia Ouardigha Doukkala Abda** | Goat | 135.995747 | 269.421585 | 18.2627711 | 92.0448085 | 99.6184805 |
| 2012 | **Taza Alhoceima Taounate Fes Boulemane** | Goat | 63.6602458 | 282.358766 | 27.1918786 | 51.8273048 | 58.0883767 |
| 2012 | **Grand Casablanca** | Goat | 4.71876745 | 17.6920698 | 0.71154315 | 2.77386437 | 6.54291246 |
| 2012 | **Laayoune Boujdour Sakia El hamra Guelmim Essmara** | Goat | 32.3929315 | 61.793528 | 0.10209171 | 10.807832 | 7.33013425 |
| 2012 | **Marrakech Tensift Al Haouz Tadla Azilal** | Goat | 119.713182 | 405.495084 | 134.294584 | 72.0402827 | 109.450804 |
| 2012 | **Oriental** | Goat | 35.4084487 | 108.147596 | 6.611322 | 17.4165758 | 29.6309539 |
| 2012 | **Rabat Sale zemmour Zaer Chrarda Bni Hssen** | Goat | 15.8836712 | 67.6799483 | 5.389366 | 11.0004506 | 15.1651278 |
| 2012 | **Souss Massa Draâ** | Goat | 169.394397 | 285.355066 | 55.2330215 | 68.4420927 | 36.1879591 |
| 2012 | **Tanger Tetouan** | Goat | 106.074984 | 321.482772 | 67.9856643 | 96.4623975 | 79.5303524 |
| 2013 | **Meknes Tafilalet** | Goat | 130.428844 | 343.427247 | 150.89503 | 97.4123851 | 121.261971 |
| 2013 | **Chaouia Ouardigha Doukkala Abda** | Goat | 129.56803 | 241.709795 | 18.6341464 | 98.0648655 | 89.4665205 |
| 2013 | **Taza Alhoceima Taounate Fes Boulemane** | Goat | 85.9922274 | 251.541945 | 12.3594977 | 70.0558438 | 68.4171143 |
| 2013 | **Grand Casablanca** | Goat | 7.0605093 | 23.6472351 | 0.8151348 | 3.4026566 | 7.86883729 |
| 2013 | **Laayoune Boujdour Sakia El hamra Guelmim Essmara** | Goat | 35.3420665 | 64.936845 | 0.33932755 | 9.09106465 | 9.3387178 |
| 2013 | **Marrakech Tensift Al Haouz Tadla Azilal** | Goat | 69.3676842 | 340.628136 | 176.065224 | 67.801118 | 131.694863 |
| 2013 | **Oriental** | Goat | 39.3620665 | 113.176116 | 4.6035382 | 15.2055462 | 34.8706558 |
| 2013 | **Rabat Sale zemmour Zaer Chrarda Bni Hssen** | Goat | 9.45649301 | 54.5274948 | 9.524844 | 9.37938952 | 18.3819627 |
| 2013 | **Souss Massa Draâ** | Goat | 165.740374 | 364.326798 | 35.4826955 | 76.6185881 | 54.4218591 |
| 2013 | **Tanger Tetouan** | Goat | 125.7121 | 310.671581 | 46.6616714 | 121.811009 | 65.9946575 |
| 2014 | **Meknes Tafilalet** | Goat | 121.017487 | 315.420469 | 143.935734 | 88.80258 | 117.138285 |
| 2014 | **Chaouia Ouardigha Doukkala Abda** | Goat | 166.559846 | 218.308795 | 28.2876664 | 106.906303 | 97.403658 |
| 2014 | **Taza Alhoceima Taounate Fes Boulemane** | Goat | 86.1101637 | 242.978513 | 41.9107978 | 87.8482579 | 74.8371396 |
| 2014 | **Grand Casablanca** | Goat | 5.14368531 | 27.2035518 | 1.07248185 | 3.81761791 | 6.24237736 |
| 2014 | **Laayoune Boujdour Sakia El hamra Guelmim Essmara** | Goat | 34.718521 | 60.361069 | 0.46073841 | 11.8291102 | 12.1768096 |
| 2014 | **Marrakech Tensift Al Haouz Tadla Azilal** | Goat | 96.6536619 | 417.074167 | 142.262039 | 74.0102048 | 146.742416 |
| 2014 | **Oriental** | Goat | 35.7051589 | 119.539957 | 6.75610723 | 12.0211758 | 49.0253722 |
| 2014 | **Rabat Sale zemmour Zaer Chrarda Bni Hssen** | Goat | 14.3049961 | 66.6855053 | 7.48446 | 16.3379044 | 17.9827202 |
| 2014 | **Souss Massa Draâ** | Goat | 165.983661 | 315.766228 | 29.2837795 | 87.3458451 | 49.1990162 |
| 2014 | **Tanger Tetouan** | Goat | 123.689109 | 317.26274 | 42.8014371 | 108.595633 | 91.5508515 |
| 2011 | **Meknes Tafilalet** | Sheep | 181.691856 | 1054.96338 | 301.898009 | 175.335255 | 371.603812 |
| 2011 | **Chaouia Ouardigha Doukkala Abda** | Sheep | 230.788675 | 440.383475 | 6.2297284 | 163.110478 | 143.615935 |
| 2011 | **Taza Alhoceima Taounate Fes Boulemane** | Sheep | 290.517355 | 1138.94853 | 109.825756 | 342.244194 | 236.314613 |
| 2011 | **Grand Casablanca** | Sheep | 170.091853 | 1078.26487 | 203.369231 | 137.031322 | 324.524939 |
| 2011 | **Laayoune Boujdour Sakia El hamra Guelmim Essmara** | Sheep | 31.9554021 | 47.7637365 | 0.048048 | 13.1403344 | 12.0857874 |
| 2011 | **Marrakech Tensift Al Haouz Tadla Azilal** | Sheep | 221.145953 | 1321.07537 | 415.982076 | 236.042688 | 498.240062 |
| 2011 | **Oriental** | Sheep | 232.175149 | 818.131788 | 47.3065344 | 150.187014 | 248.971886 |
| 2011 | **Rabat Sale zemmour Zaer Chrarda Bni Hssen** | Sheep | 142.13263 | 704.90318 | 71.1516257 | 167.38652 | 157.809342 |
| 2011 | **Souss Massa Draâ** | Sheep | 183.372381 | 267.981487 | 36.2036523 | 102.951669 | 50.4579773 |
| 2011 | **Tanger Tetouan** | Sheep | 92.8139203 | 384.236494 | 31.6872081 | 109.761059 | 78.1474455 |
| 2012 | **Meknes Tafilalet** | Sheep | 192.430492 | 989.66273 | 358.737169 | 228.720652 | 380.803071 |
| 2012 | **Chaouia Ouardigha Doukkala Abda** | Sheep | 219.203125 | 468.0407 | 25.3319347 | 177.883231 | 144.337948 |
| 2012 | **Taza Alhoceima Taounate Fes Boulemane** | Sheep | 287.352501 | 1087.07878 | 101.910647 | 375.844637 | 282.105089 |
| 2012 | **Grand Casablanca** | Sheep | 137.209107 | 1127.73924 | 59.1699118 | 64.0594175 | 455.418893 |
| 2012 | **Laayoune Boujdour Sakia El hamra Guelmim Essmara** | Sheep | 34.9356895 | 65.178261 | 0.41850463 | 16.1499452 | 9.2730978 |
| 2012 | **Marrakech Tensift Al Haouz Tadla Azilal** | Sheep | 220.50559 | 1374.81591 | 443.0058 | 186.216364 | 495.1856 |
| 2012 | **Oriental** | Sheep | 347.894999 | 818.077337 | 44.5556322 | 169.727024 | 218.393687 |
| 2012 | **Rabat Sale zemmour Zaer Chrarda Bni Hssen** | Sheep | 121.587393 | 681.160057 | 45.4041403 | 121.137692 | 195.302049 |
| 2012 | **Souss Massa Draâ** | Sheep | 194.436054 | 366.446503 | 55.57905 | 75.852891 | 45.9941835 |
| 2012 | **Tanger Tetouan** | Sheep | 99.432588 | 377.385874 | 52.7811167 | 116.211653 | 83.6063336 |
| 2013 | **Meknes Tafilalet** | Sheep | 207.469183 | 1005.70258 | 375.662185 | 238.084271 | 365.421191 |
| 2013 | **Chaouia Ouardigha Doukkala Abda** | Sheep | 227.50742 | 465.027935 | 12.2924663 | 182.851533 | 154.083881 |
| 2013 | **Taza Alhoceima Taounate Fes Boulemane** | Sheep | 326.86379 | 1029.87168 | 83.5876864 | 388.021877 | 267.618288 |
| 2013 | **Grand Casablanca** | Sheep | 161.47828 | 1061.25919 | 91.6966911 | 140.753253 | 373.5947 |
| 2013 | **Laayoune Boujdour Sakia El hamra Guelmim Essmara** | Sheep | 42.0945727 | 69.0367415 | 0.50247 | 13.9708861 | 13.3364369 |
| 2013 | **Marrakech Tensift Al Haouz Tadla Azilal** | Sheep | 208.893208 | 1342.59663 | 440.208696 | 207.547127 | 500.991451 |
| 2013 | **Oriental** | Sheep | 297.897023 | 970.09965 | 31.531395 | 162.478557 | 291.096223 |
| 2013 | **Rabat Sale zemmour Zaer Chrarda Bni Hssen** | Sheep | 117.817598 | 639.951574 | 78.0088059 | 148.970225 | 196.800058 |
| 2013 | **Souss Massa Draâ** | Sheep | 187.28737 | 387.07847 | 38.257108 | 83.9814319 | 52.2891666 |
| 2013 | **Tanger Tetouan** | Sheep | 122.05452 | 391.059837 | 45.2535025 | 140.670777 | 78.7442587 |
| 2014 | **Meknes Tafilalet** | Sheep | 211.994655 | 922.416127 | 352.717367 | 201.254824 | 384.832822 |
| 2014 | **Chaouia Ouardigha Doukkala Abda** | Sheep | 232.233425 | 408.890733 | 12.5242204 | 169.64571 | 211.984384 |
| 2014 | **Taza Alhoceima Taounate Fes Boulemane** | Sheep | 331.563324 | 1069.79565 | 146.724099 | 366.348283 | 304.916864 |
| 2014 | **Grand Casablanca** | Sheep | 139.623259 | 1039.1479 | 167.544351 | 103.571621 | 454.442988 |
| 2014 | **Laayoune Boujdour Sakia El hamra Guelmim Essmara** | Sheep | 43.533012 | 74.2520915 | 0.36555136 | 20.5319435 | 15.4442208 |
| 2014 | **Marrakech Tensift Al Haouz Tadla Azilal** | Sheep | 208.446831 | 1414.57465 | 484.014312 | 224.745905 | 460.877076 |
| 2014 | **Oriental** | Sheep | 244.434199 | 955.267775 | 27.413762 | 95.3317268 | 383.777442 |
| 2014 | **Rabat Sale zemmour Zaer Chrarda Bni Hssen** | Sheep | 139.326962 | 627.160263 | 86.824598 | 154.30516 | 182.20384 |
| 2014 | **Souss Massa Draâ** | Sheep | 185.956715 | 382.60296 | 31.5179722 | 85.950942 | 59.5091944 |
| 2014 | **Tanger Tetouan** | Sheep | 139.759593 | 371.381746 | 46.8414414 | 132.360322 | 105.059136 |
