# Supplementary material for: The socio-economic burden of cystic echinococcosis in Morocco: A combination of estimation method
Source: PLoS Negl Trop Dis. 2020 Jul 31;14(7):e0008410. doi: 10.1371/journal.pntd.0008410 (PMC7423152; doi:10.1371/journal.pntd.0008410)
Supplement: S9 Table — (DOCX) [file pntd.0008410.s009.docx]

- Table S9: Number of cows by region and by year (per 1000 heads). Source: HCP

|  |  | **< 1**  **years old** | **1 à <2 years old** | **2 à <3 years old** | **3 à < years old** | **+9**  **years old** |
| --- | --- | --- | --- | --- | --- | --- |
| 2011 | **Meknes Tafilalet** | 52.874715 | 40.3614776 | 15.3147074 | 145.186844 | 12.8619797 |
| 2011 | **Chaouia Ouardigha Doukkala Abda** | 8.59760844 | 7.68117454 | 12.3773861 | 48.6186871 | 0.41839865 |
| 2011 | **Taza Alhoceima Taounate Fes Boulemane** | 25.7694256 | 33.7102283 | 47.3000295 | 144.479004 | 8.77454789 |
| 2011 | **Grand Casablanca** | 74.8421363 | 38.2627261 | 45.2672829 | 221.447838 | 23.5221735 |
| 2011 | **Laayoune Boujdour Sakia El hamra Guelmim Essmara** | 0.432 | 0.514 | 1.0485 | 0.619 | 0 |
| 2011 | **Marrakech Tensift Al Haouz Tadla Azilal** | 78.8203375 | 39.3616415 | 48.1958604 | 198.983569 | 17.145486 |
| 2011 | **Oriental** | 11.6643597 | 6.16534627 | 8.29483519 | 33.8203067 | 1.48281924 |
| 2011 | **Rabat Sale zemmour Zaer Chrarda Bni Hssen** | 56.5181521 | 39.3860822 | 44.361464 | 200.91256 | 11.1482952 |
| 2011 | **Souss Massa Draâ** | 16.957152 | 24.6791304 | 27.207205 | 86.6402764 | 0 |
| 2011 | **Tanger Tetouan** | 48.9849421 | 47.3141644 | 42.764235 | 196.794409 | 10.6051743 |
| 2012 | **Meknes Tafilalet** | 58.7640594 | 44.2437549 | 15.6023536 | 158.042289 | 21.7254335 |
| 2012 | **Chaouia Ouardigha Doukkala Abda** | 9.29376907 | 7.21387463 | 11.5296744 | 46.212098 | 2.97785173 |
| 2012 | **Taza Alhoceima Taounate Fes Boulemane** | 28.4172986 | 35.4807208 | 50.9743347 | 141.914039 | 11.3282339 |
| 2012 | **Grand Casablanca** | 98.3677084 | 29.355007 | 40.5693415 | 235.227005 | 26.7796534 |
| 2012 | **Laayoune Boujdour Sakia El hamra Guelmim Essmara** | 0.23 | 0.47 | 0.7885 | 0.81 | 0 |
| 2012 | **Marrakech Tensift Al Haouz Tadla Azilal** | 91.0800535 | 33.2750952 | 30.0319119 | 197.006095 | 45.591712 |
| 2012 | **Oriental** | 11.4551573 | 8.07099015 | 8.0375386 | 32.2100718 | 2.77344525 |
| 2012 | **Rabat Sale zemmour Zaer Chrarda Bni Hssen** | 59.8616631 | 28.2646247 | 39.6448167 | 196.652527 | 11.8925855 |
| 2012 | **Souss Massa Draâ** | 14.0798418 | 22.2234244 | 26.3144087 | 89.3083159 | 5.74721163 |
| 2012 | **Tanger Tetouan** | 57.4311802 | 43.7537113 | 47.2529892 | 200.87337 | 17.5403103 |
| 2013 | **Meknes Tafilalet** | 57.787261 | 75.3789858 | 17.1588035 | 167.681316 | 23.0832081 |
| 2013 | **Chaouia Ouardigha Doukkala Abda** | 8.07348186 | 6.20596594 | 8.82553202 | 46.3001932 | 1.59935055 |
| 2013 | **Taza Alhoceima Taounate Fes Boulemane** | 28.4186768 | 34.1824357 | 48.4411028 | 148.640386 | 11.5518773 |
| 2013 | **Grand Casablanca** | 98.6928681 | 37.6669164 | 54.7339352 | 234.050734 | 30.8727947 |
| 2013 | **Laayoune Boujdour Sakia El hamra Guelmim Essmara** | 0.251 | 0.23 | 0.6115 | 0.7885 | 0 |
| 2013 | **Marrakech Tensift Al Haouz Tadla Azilal** | 86.2711459 | 33.0411134 | 29.1246271 | 173.832266 | 87.3347373 |
| 2013 | **Oriental** | 12.1085 | 6.7805 | 8.2235 | 32.9455 | 2.6135 |
| 2013 | **Rabat Sale zemmour Zaer Chrarda Bni Hssen** | 67.0497542 | 39.2248873 | 31.851472 | 201.056493 | 21.0248096 |
| 2013 | **Souss Massa Draâ** | 12.9968262 | 25.0420897 | 27.3130087 | 89.0707579 | 3.65456795 |
| 2013 | **Tanger Tetouan** | 54.5043331 | 43.1546178 | 45.7146174 | 187.711422 | 19.3104939 |
| 2014 | **Meknes Tafilalet** | 44.5339772 | 21.4543476 | 40.5458803 | 108.585934 | 16.6445557 |
| 2014 | **Chaouia Ouardigha Doukkala Abda** | 6.56876435 | 5.89551176 | 7.55568462 | 49.1441052 | 2.76198 |
| 2014 | **Taza Alhoceima Taounate Fes Boulemane** | 36.9522446 | 30.0131302 | 49.6036238 | 142.365318 | 17.2253865 |
| 2014 | **Grand Casablanca** | 110.883008 | 28.02124 | 44.7346314 | 227.253924 | 24.2528896 |
| 2014 | **Laayoune Boujdour Sakia El hamra Guelmim Essmara** | 0.29378044 | 0.22121316 | 0.73269543 | 0.83218285 | 0 |
| 2014 | **Marrakech Tensift Al Haouz Tadla Azilal** | 83.8542078 | 37.1763521 | 31.2605854 | 196.192013 | 84.2439754 |
| 2014 | **Oriental** | 14.2374315 | 6.40394802 | 6.81636301 | 36.1100364 | 1.33028929 |
| 2014 | **Rabat Sale zemmour Zaer Chrarda Bni Hssen** | 63.2853192 | 41.9802752 | 31.9844184 | 194.408251 | 18.5595393 |
| 2014 | **Souss Massa Draâ** | 11.7385264 | 24.3371639 | 36.9107728 | 95.4466525 | 3.29984806 |
| 2014 | **Tanger Tetouan** | 57.8827891 | 36.2686035 | 48.6242654 | 184.212206 | 14.8877008 |
